# TAZ promotes osteogenic differentiation of mesenchymal stem cells line C3H10T1/2, murine multi-lineage cells lines C2C12, and MEFs induced by BMP9

**DOI:** 10.1038/s41420-022-01292-y

**Published:** 2022-12-27

**Authors:** Huakun Huang, Qiuping Lu, Caihong Ye, Mengqi Wei, Chunmei Yang, Lulu Zhang, Yanran Huang, Xiaoji Luo, Jinyong Luo

**Affiliations:** 1 Key Laboratory of Clinical Laboratory Diagnostics, Ministry of Education, Chongqing Medical University, 400016 Chongqing, China; 2 Department of Orthopedics, The First Affiliated Hospital of Chongqing Medical University, 400016 Chongqing, China

**Keywords:** Mesenchymal stem cells, Phosphoinositol signalling

## Abstract

Bone morphogenetic protein 9 (BMP9), also named as growth differentiation factor 2 (GDF-2), is the strongest cytokine that promotes osteogenic differentiation in the BMP family, and has broad clinical application value. Nevertheless, the mechanism of BMP9 promotes osteogenic differentiation remain unclear. TAZ, a transcriptional co-activator, has great effects on cell proliferation, differentiation, and stem cell self-renewal. In this research, we investigated the effects of TAZ in BMP9-induced osteogenic differentiation of mesenchymal stem cell line C3H10T1/2 (MSCs) and murine multi-lineage cell lines C2C12 and MEFs (MMCs) and explored its possible mechanisms. This study has found that BMP9 induces the expression of TAZ and promotes its nuclear translocation. Meanwhile, our study found that Ad-TAZ and TM-25659, a TAZ agonist, can enhance the osteogenic differentiation of MSCs and MMCs induced by BMP9. Conversely, Ad-si-TAZ and verteporfin, an inhibitor of TAZ, have the contradictory effect. Likewise, the promotion of TAZ to the BMP9-induced ectopic bone formation in vivo was confirmed by the subcutaneous transplantation of MSCs in nude mice. Furthermore, we have detected that TAZ might increase the levels of the phosphorylation of Smad1/5/8, p38, ERK1/2, and JNK induced by BMP9. Additionally, we also found that TAZ increased the total protein level of β-catenin induced by BMP9. In summary, our results strongly indicated that TAZ will promote the osteogenic differentiation in MSCs and MMCs induced by BMP9 through multiple signal pathways.

## Introduction

Due to various factor, such as fractures, bone defects, nonunion, severe trauma, bone tumor resection, and population aging, the demand for bone repairability is increasing rapidly, which is one of the most challenging clinical treatments [1–3]. Autologous bone transplantation has long been considered the gold standard for bone transplantation. However, autogenous bone transplantation will probably lead to a series of problems, including chronic pain, infection, high cost of prolonged surgery, additional incision for bone extraction, and most importantly, limited autogenous bone source [4–6]. Bone tissue engineering based on three-dimensional scaffolds in combination with cytokines and MSCs, which have been confirmed to promote the repairability of fractures and bone defects, by providing better technical support for clinical treatment [7, 8]. MSCs are multi-directional differentiated functional stem cells, which differentiate into a variety of cells under different induction conditions, including adipocytes, chondrocytes, and osteoblasts [9–11]. Moreover, MSCs are widely recommended for the tissue engineering due to the large differentiation potential, low immunogenicity, strong self-renewal capability, and extensive sources [12–14].

BMP9 also named as GDF-2, is a member of the transforming growth factor (TGF)/BMP superfamily, which was first discovered in the liver of mice [15–17]. It has been reported that BMP9 plays a role in promoting cutaneous wound healing [18], regulating glucose metabolism [19, 20], improving amyloidosis, cholinergic deficiency, pathology, and cognitive deficiencies in a mouse model of Alzheimer’s disease [21, 22]. At present, more than 20 BMPs have been confirmed, and of which, BMP2, BMP6, and BMP7 have been verified to play a crucial role in the osteogenic differentiation of MSCs [23]. Furthermore, BMP2 and BMP7 have been evaluated for clinical treatment of tibial fractures and spinal fusion [24–26]. However, the analysis of clinical reports showed that the curative effect of BMP2 and BMP7 were not satisfactory, which might lead to the cystic bone and bone density decreases [27, 28]. In view of these uncertainties, a more powerful osteogenic inducer is needed indeed in the clinical treatment. A lot of studies have reported that BMP9, as the strongest bone-promoting factor among BMPs, will promote the osteogenic differentiation of MSCs [29, 30]. When BMP9 binds to the receptors BMPR1 and BMPR2 to form a heterotetrameric complex, the intracellular domain of BMPR1 subsequently initiates Smad1/5/8 phosphorylation [31, 32]. Phosphorylated Smad1/5/8 and synergistic Smad (co-Smad, Smad4) combine to form a Smad1/5/8-Smad complex, which is finally transported into the nucleus to initiate the transcription of downstream osteogenic-related genes such as Id1 (inhibitor of differentiation 1), Id2 (inhibitor of differentiation 2), Id3 (inhibitor of differentiation 3), RUNX2 (runt-related transcription factor 2), DLX5 (Distal-less homeobox 5), and OSX (Osterix) [32, 33]. Despite these valuable findings, BMP9 remains the least studied BMPs. Therefore, it is necessary to understand the regulatory mechanisms behind the MSCs differentiation, which was regulated by BMP9 to improve the clinical application of BMP9. It has been reported that other cytokines combined with BMP9 can promote osteogenic differentiation of MSCs induced by BMP9, which will further optimize the bone-promoting effect of BMP9 [34, 35]. Nevertheless, the exact biological molecular mechanism involved is still under investigation.

TAZ (also known as WWTR1), a transcription co-activator with PDZ-binding motif, is an essential downstream factor of the Hippo signaling pathway. It regulates cell proliferation, apoptosis, and stem cell differentiation through multiple signaling pathways, such as the signaling pathways of Wnt/β-catenin, NF-kB, and Hippo [36–38]. The MST and LATS kinases undergo a series of phosphorylation reactions, when the Hippo signaling pathway is activated, and eventually lead to the phosphorylation of the key effector TAZ. The transcription of TAZ will be inhibited when phosphorylated TAZ was degraded in the cytoplasm by ubiquitination [39, 40]. On the contrary, the inactivation of the Hippo signaling pathway will increase the nuclear translocation of TAZ and promotes downstream transcription [41]. A great many reports recently have demonstrated that TAZ is required for chondrogenesis and skeletal development [42, 43], and inhibited adipogenic differentiation of MSCs [44], which indicted suggesting that TAZ might play the central role in the skeleton-genesis and bone regeneration. Nevertheless, TAZ has synergistic effect with BMP9 and its mechanism which have not been reported until now. Hence, we sought to explore the role of TAZ in the osteogenic differentiation of MSCs and MMCs induced by BMP9. This study found that BMP9 could induce the expression of TAZ and promote its nuclear translocation in the MSCs and MMCs. Furthermore, the result of chromatin immunoprecipitation (ChIP) assay showed that BMP9 induces the recruitment of Smad1/5/8 to the promoter of TAZ, which suggested that TAZ might be a direct target molecule for BMP/Smad signaling. Meanwhile, we also found that Ad-TAZ and TM-25659 were performed to promote the osteogenic differentiation of BMP9-induced MSCs and MMCs; conversely, Ad-si-TAZ and verteporfin have the opposite effect. Moreover, we have confirmed that TAZ might increase the levels of the phosphorylation of Smad1/5/8, p38, ERK1/2, and JNK which was induced by BMP9. Additionally, TAZ would increase the total protein level of β-catenin induced by BMP9.

In summary, our results strongly indicated that TAZ would promotes the osteogenic differentiation in MSCs and MMCs induced by BMP9 through multiple signaling pathways.

## Results

### The expression of TAZ was induced by BMP9 and its nuclear translocation in MSCs and MMCs was promoted

**Fig. 1:**
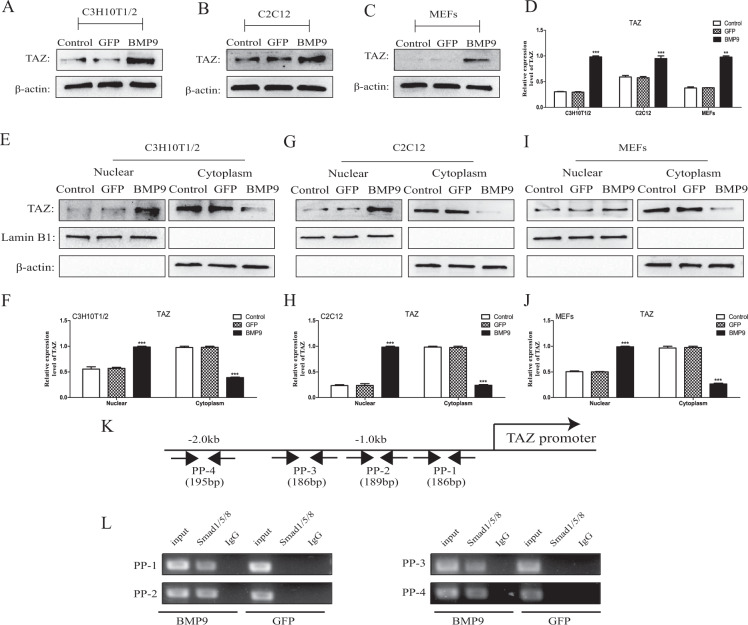
The effects of BMP9 on TAZ expression in MSCs and MMCs, TAZ was a direct target of BMP9/Smad signaling. **A**–**D** The protein expression level of TAZ was detected by Western blot after Ad-BMP9 or Ad-GFP infection for 24 h. **E**–**J** The nucleoprotein and cytoplasmic protein expression levels of TAZ were detected by western blot after Ad-BMP9 or Ad-GFP infection for 24 h. **K** Prediction of Smad1/5/8 binding sites in the TAZ promoter region. **L** Enrichment of Smad1/5/8 on the PP-1, PP-2, PP-3, and PP-4 fragment of the TAZ promoter were measured by ChIP in MSCs. The data were shown as mean ± SD for three separated experiments. (*P < 0.05, **P < 0.01, ***P < 0.001) MSCs mesenchymal stem cells, MMCs murine multi-lineage cells, ChIP chromatin immunoprecipitation, Ad-GFP adenovirus carrying green fluorescent protein gene, Ad-BMP9 adenovirus carrying bone morphogenetic protein 9 gene, IgG immunoglobulin G, GAPDH, glyceraldehyde 3-phosphate-dehydrogenase, TAZ transcriptional co-activator with PDZ-binding motif.

The interaction between BMP9 and TAZ remains still unclear in MSCs and MMCs, so that, we intend to investigate the effects in this research. First of all, by western blot we have found that BMP9 would increase the protein level of TAZ in MSCs and MMCs (Fig. 1A–D). Previous studies have shown that TAZ plays a signal transduction role mainly in the nucleus [41]. Consequently, we have planned to explore whether BMP9 promotes the TAZ nuclear translocation. The results of analyzed by western blot (Fig. 1E–J) confirmed that BMP9 could promote TAZ nuclear translocation. These results suggested that BMP9 effectively increases the expression of TAZ and promotes its nuclear translocation in MSCs and MMCs.

### TAZ is a direct target molecule for BMP9/Smad signaling

Our results have confirmed that BMP9 could promote the expression of TAZ. Next, we aimed to analyze whether the BMP9-specific transcription factor Smad1/5/8 binds to the TAZ promoter region to verify TAZ is a direct target of BMP9/Smad signaling. Consequently, the ChIP experiment to detect that Smad1/5/8 was enriched conducted in the TAZ promoter region in this study. Four pairs of primers were designed upstream of the TAZ promoter to detect sites where TAZ might bind to BMP R-Smad (Fig. 1K), determined by semi-quantitative PCR analysis. As expected, the Smad1/5/8 binding to TAZ promoter was induced by the stimulation of BMP9 (Fig. 1L). The above results indicated that TAZ was regulated by BMP9 with its specific transcription factor Smad1/5/8.

### Effects of TAZ on early and late osteogenic differentiation induced by BMP9

**Fig. 2:**
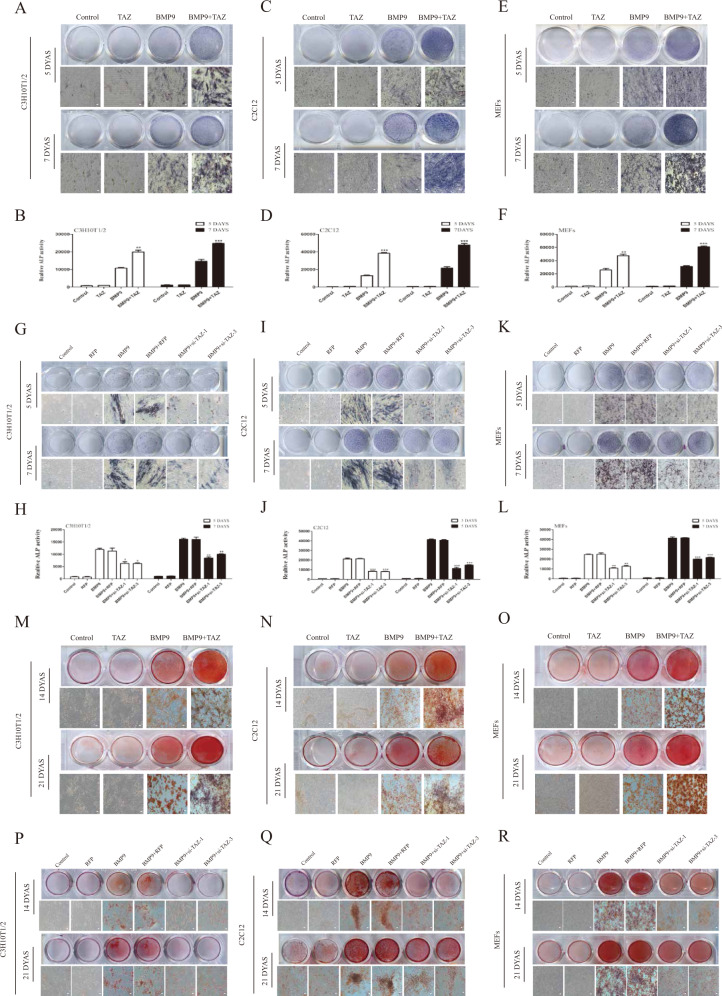
The effect of TAZ on BMP9-induced early and late osteogenic differentiation in MSCs and MMCs. Cells were treated with TAZ or si-TAZ, followed by treatment with BMP9. **A**–**L** The ALP activity was performed by chemiluminescence assay and ALP staining was conducted by histochemical staining assay at 5 and 7 days. **M**–**R** Calcium deposition was analyzed by Alizarin Red S staining at 14 and 21 days. The data were shown as mean ± SD for three separate experiments. Ad-RFP adenovirus carrying red fluorescent protein gene.

We found that TAZ was regulated by BMP9 with its specific transcription factor Smad1/5/8. This finding prompted us to explore the effects of TAZ on the osteogenic differentiation induced by BMP9. Therefore, we intended to explore the effects of TAZ on the early and late osteogenic differentiation induced by BMP9. Alkaline phosphatase (ALP) assay was a good indicator of early osteogenic differentiation. Correspondingly, Alizarin Red S Staining was used to detect changes in calcium deposition, one of the late osteogenic indicators. The Ad-TAZ and Ad-si-TAZ recombinant adenovirus was constructed in this trial and the effectiveness of Ad-TAZ and Ad-si-TAZ were verified by western blot (Fig. S1A–H). Besides, by ALP staining and ALP activity (Fig. S1I–T) we found that TM-25659 and verteporfin themselves had no effect on the osteogenic differentiation of MSCs and MMCs. And cells were treated by various treatments in compliance with the experimental design. We have found that ALP staining, ALP activity and calcium deposition were increased in the BMP9 + TAZ group and BMP9 + TM-25659 group compared to BMP9 group (Fig. 2A–F, M–O) (Fig. S2A–F, M–O), which suggested that the osteogenic differentiation potential of MSCs and MMCs were increased. However, impaired ALP activity, lower ALP staining and calcium deposition were detected in the BMP9 + si-TAZ group and BMP9 + verteporfin group compared to BMP9 group (Fig. 2G–L, P–R) (Fig. S2G–L, P–R). These results suggested that Ad-TAZ and TM-25659 can promote the early and late osteogenic differentiation of MSCs and MMCs that induced by BMP9, while the results of Ad-si-TAZ and verteporfin were opposite to those of the Ad-TAZ.

### Effects of TAZ on the expression of osteogenic-related factors induced by BMP9

**Fig. 3:**
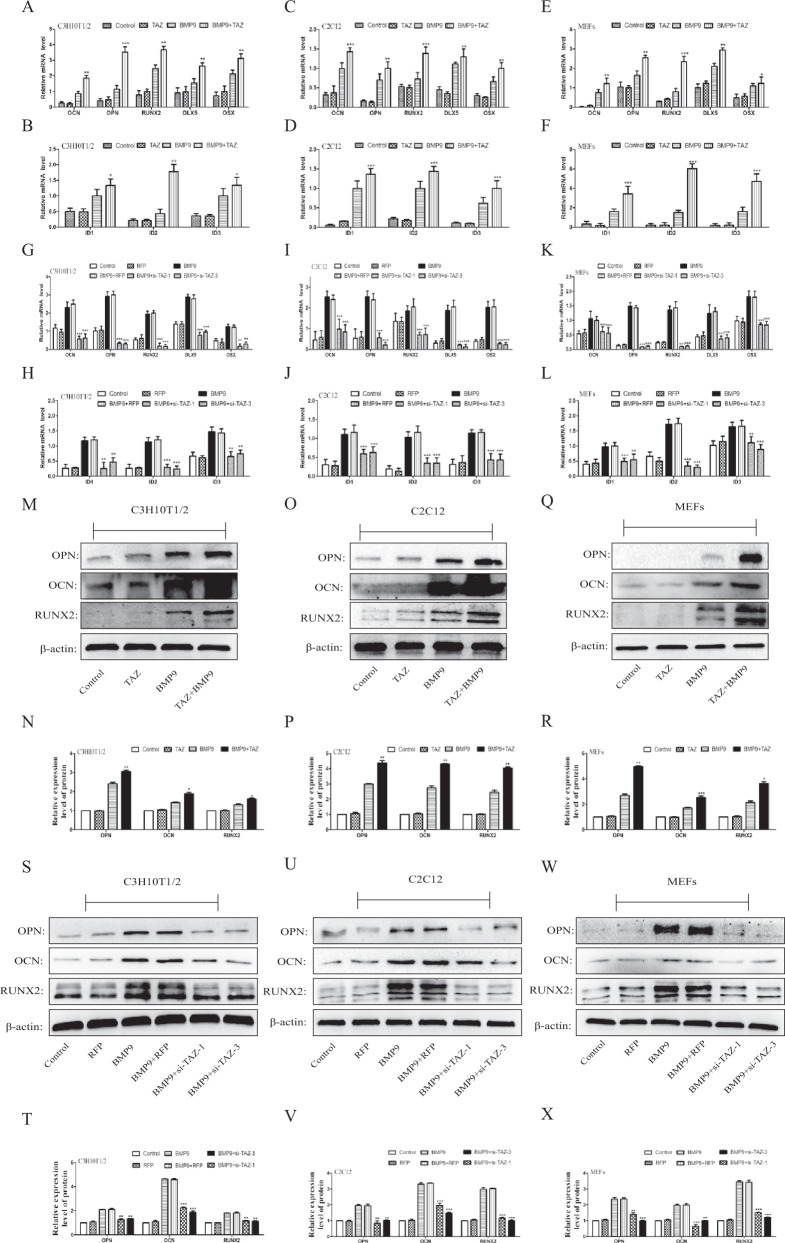
The effect of TAZ on BMP9-induced expression levels of pivotal osteogenic markers in MSCs and MMCs. Cells were treated with TAZ or si-TAZ, followed by treatment with BMP9. **A**–**L** The mRNA expression of RUNX2, OPN, OCN, DLX5, ID1, ID2, and ID3 were determined by qPCR. **M**–**X** The protein expression of RUNX2, OPN and OCN were detected by Western blot. The data are shown as mean ± SD for three separated experiments. (*P < 0.05, **P < 0.01, ***P < 0.001) RUNX2 runt-related transcription factor 2, OCN osteocalcin, OPN osteopontin, DLX5 Distal-less homeobox 5, OSX Osterix, ID Inhibitor of differentiation.

A great number of studies have confirmed that osteopontin (OPN), and osteocalcin (OCN), RUNX2 are key indicators to the osteogenic differentiation of MSCs [32, 33]. And we investigated the effects of TAZ on the vital indicators for osteogenic differentiation which induced by BMP9 with using qRT-PCR analysis and western blot. Our results revealed that the mRNA levels (Fig. 3A–F) (Fig. S3A–F) and protein levels (Fig. 3M–R) (Fig. S3M–R) of osteogenic-related factors were increased obviously in the BMP9 + TAZ group and BMP9 + TM-25659 group, compared with the BMP9 group. In contrast, the mRNA levels (Fig. 3G–L) (Fig. S3G–L) and protein levels (Fig. 3S–X) (Fig. S3S–X) of osteogenic-related factors were lower in the BMP9 + si-TAZ group and BMP9 + verteporfin group than in the BMP9 group. The above results suggested that TAZ can promote the BMP9-induced osteogenic differentiation of MSCs and MMCs.

### TAZ promoted BMP9-induced ectopic bone transplantation with MSCs

**Fig. 4:**
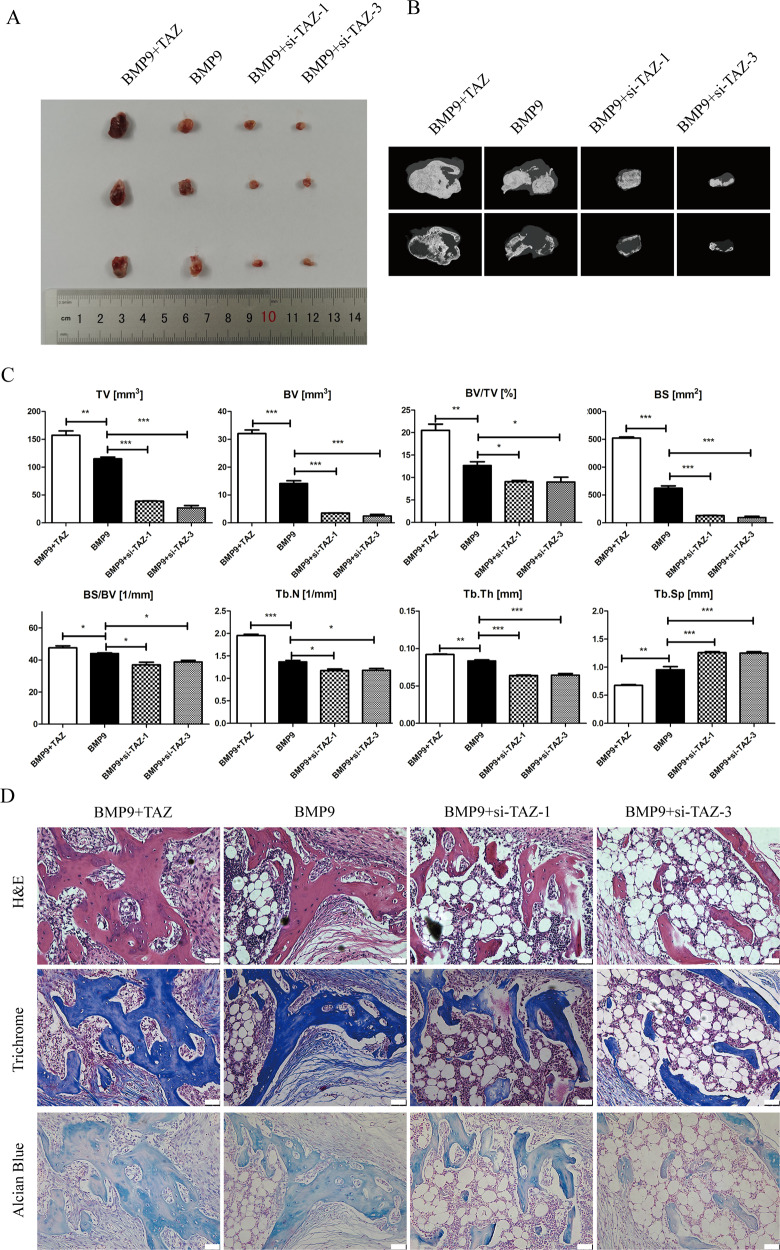
The effect of TAZ on BMP9-induced ectopic bone formation in MSCs and MMCs. **A** The general observation of the subcutaneous mass of ectopic osteogenesis in nude mice. **B** Subcutaneous osteoblast mass for micro-CT scanning to get a representative reconstructed 3D image. **C** Quantitative analysis of bone tissue and the values of TV, BV, BV/TV, BS, BS/BV, Tb.N, Tb.Th, and Tb.Sp were analyzed. **D** H&E staining and Masson’s Trichrome staining and Alcian Blue staining to detect the formation of trabecular bone, bone matrix, and cartilage matrix under the treatment as shown. The data were shown as mean ± SD for three separated experiments. (*P < 0.05, **P < 0.01, ***P < 0.001). TV tissue volume, BV bone volume, BV/TV percent bone volume, BS bone surface, BS/BV bone surface/volume ratio, Tb.N trabecular number, Tb.Th trabecular thickness, Tb.Sp trabecular separation.

Now that the osteogenic differentiation of BMP9-induced MSCs in vitro can be promoted by TAZ, which have been confirmed, we would like to further determine the effects of TAZ on the osteogenic differentiation of MSCs induced by BMP9 in vivo. Meanwhile, we have used the MSCs to simulate ectopic bone formation. Compared with the BMP9 group, the general observation and the results of micro-CT three-dimensional (3D) reconstruction showed that the total bone volume was increased in the BMP9 + TAZ group, while decreased in the BMP9 + si-TAZ group (Fig. 4A, B). Compared with the BMP9 group, further quantitative analysis of bone histomorphology revealed that the values of TV, BV, BV/TV, BS, BS/BV, Tb.N, and Tb.Th were increased while Tb.Sp was decreased in the BMP9 + TAZ group. The results of the BMP9 + si-TAZ group were opposite to those of the BMP9 + TAZ group (Fig. 4C). H&E staining and Masson Trichrome staining (Fig. 4D) showed that compared the quality, quantity and structural integrity of trabecular bone in the BMP9 + TAZ group were increased, compared with the BMP9 group, while decreased in the BMP9 + si-TAZ group. The Alcian Blue staining (Fig. 4D) have further confirmed the quantity of cartilage matrix in the BMP9 + TAZ group were increased, compared with the BMP9 group, while decreased in the BMP9 + si-TAZ group. Therefore, the above results confirmed that TAZ can promote the osteogenic differentiation of BMP9-induced MSCs in vitro and in vivo.

### Effects of TAZ on classical Smad1/5/8 signaling, Wnt/β-catenin signaling, and MAPKs signaling induced by BMP9

**Fig. 5:**
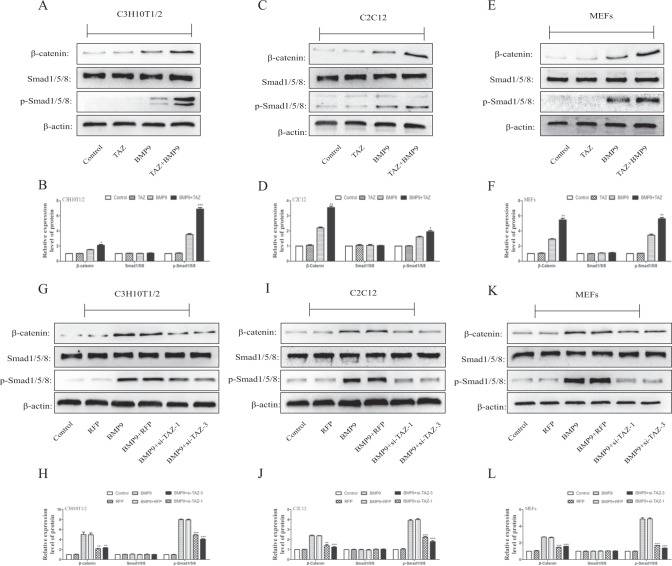
The effect of TAZ on BMP9-induced classical Smad1/5/8 and Wnt/β-catenin in MSCs and MMCs. Cells were treated with TAZ or si-TAZ, followed by treatment with BMP9. **A**–**L** The total amount and phosphorylated forms of Smad1/5/8 were measured by western blot. **A**–**L** The total amount of β-catenin was measured by western blot. The data were shown as mean ± SD for three separated experiments. (*P < 0.05, **P < 0.01, ***P < 0.001).

Next, we attempted to investigate the possible molecular mechanism of TAZ on BMP9-induced osteogenic differentiation, and tried to further explain the possible mechanisms. Previous studies have showed that BMP9 have promoted the osteogenic differentiation of MSCs through the classical Smad1/5/8 and Wnt/β-catenin signaling pathways [32, 33, 45]. On that account, we attempted to explore that TAZ might have the possibilities to regulate the BMP9-induced osteogenic differentiation of MSCs and MMCs by affecting these signaling pathways. Using the western blot, we have found that the level of phosphorylation of Smad1/5/8 and the total protein level of β-catenin were higher in the BMP9 + TAZ group and BMP9 + TM-25659 group (Fig. 5A–F) (Fig. S4A–F) and lower in the BMP9 + si-TAZ group and BMP9 + verteporfin group (Fig. 5G–L) (Fig. S4G–L) than in the BMP9 group. In conclusion, the BMP9-induced osteogenic differentiation of MSCs and MMCs was indeed promoted by TAZ through the classical Smad1/5/8 and Wnt/β-catenin signaling pathways.

**Fig. 6:**
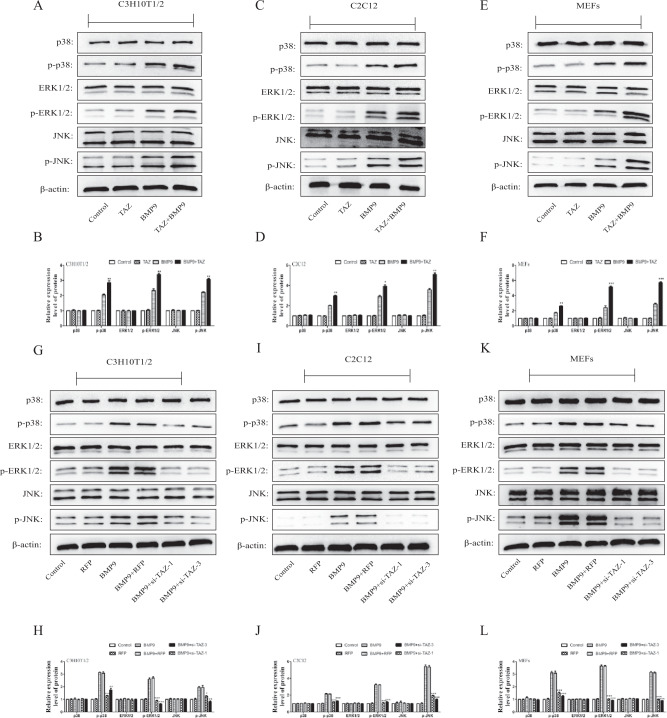
The effect of TAZ on BMP9-induced MAPKs in MSCs and MMCs. Cells were treated with TAZ or si-TAZ, followed by treatment with BMP9. **A**–**L** The total amount of p38, ERK1/2, and JNK and the phosphorylated forms of p38, ERK1/2, and JNK were measured by western blot. The data were shown as mean ± SD for three separated experiments. (*P < 0.05, **P < 0.01, ***P < 0.001).

At the same time, recent studies have showed that BMP9 can promote the osteogenic differentiation of MSCs through MAPKs signaling pathways [46, 47]. These observations gave us stimulus to explore that MAPKs signaling pathways are probably involved in the regulation of TAZ in regulates the osteogenic differentiation of MSCs and MMCs that induced by BMP9. The results of western blot showed that levels of phosphorylation of p38, JNK and ERK1/2 were higher in the BMP9 + TAZ group and BMP9 + TM-25659 group (Fig. 6A–F) (Fig. S5A–F) and lower in the BMP9 + si-TAZ group BMP9 + verteporfin group (Fig. 6G–L) (Fig. S5G–L) than in the BMP9 group. Taken together, these results indicated TAZ could promote the osteogenic differentiation of MSCs and MMCs induced by BMP9 partly through the activation of MAPKs signaling pathways.

## Discussion

BMP, discovered by Marshall Ulster in 1965, is one of the key cytokines for inducing osteogenic differentiation in bone regeneration [48]. Additionally, BMPs play a remarkable important regulatory role in cell proliferation and differentiation during embryonic development [49]; so far, at least 20 BMPs have been identified. Among them, BMP9 is the strongest bone-promoting factor in BMP family, which might play a central role in regulating lineage commitment and terminal differentiation of MSCs [29, 30]. This draws our attention to that of the BMP9 might become a potential drug for the treatment of bone diseases in the future. To explore the regulatory mechanism of BMP9-induced osteogenic differentiation of MSCs, which may be beneficial for human to further understand and study the pathogenesis of bone diseases. Meanwhile, there are more effective strategies offered by its potential mechanisms for conducting the early application of BMP9 in bone tissue engineering.

TAZ, a transcriptional co-activator, regulates the differentiation of stem cells as well as bone maintenance and development by interacting with a variety of transcription factors [36–38]. Besides, TAZ still makes great adjustments in the cell proliferation, epithelial-mesenchymal cell transformation, and osteoarthritis cartilage degradation [41, 50]. It is reported that TAZ performed the induction of the transcriptional regulation to further regulate cytoskeletal dynamics and ensure appropriate angiogenic responses, which might promote the angiogenesis and development [51]. Several studies have revealed that insulin‐like growth fac‐tor 1 (IGF‐1) and GLP‐1 receptor agonist (GLP‐1RA) can promote osteoblastogenesis by increasing TAZ expression [52, 53]. Meanwhile, it has reported that Sal B can promote the osteogenic differentiation of MSCs by upregulating the expression of TAZ [44]. The role of TAZ in skeletal development has been widely and fully studied recently; therefore, whether TAZ has synergistic effect with BMP9 in osteogenic differentiation of MSCs needs an answer.

**Fig. 7:**
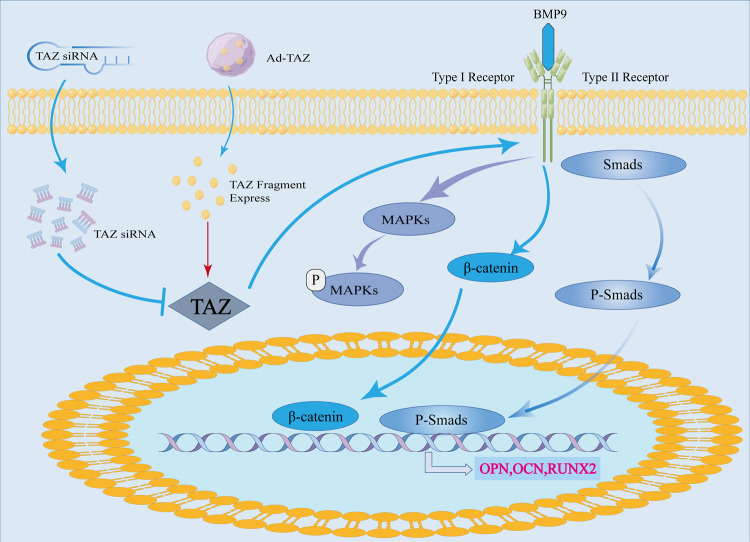
Schematic illustration of TAZ in MSCs and its mechanism of interaction with BMP/Smad, MAPKs, and Wnt/β-catenin signaling. The BMP pathway is activated by BMP9. BMPR-I activates Smads and phosphorylates Smad to form a Smad1/5/8-Smad complex, which is eventually transported into the nucleus and to initiates the transcription of downstream osteogenic-related genes. Meanwhile, BMP pathway promotes β-catenin expression and MAPK signaling pathway phosphorylation. The expression Ad-TAZ recombinant adenovirus produces TAZ and promotes classical Smad1/5/8, Wnt/β-catenin, and MAPKs signaling pathways induced by BMP9.

Our study investigated the effects of TAZ in BMP9 by regulating osteogenic of MSCs and MMCs and its potential mechanisms. We found that BMP9 would increase the expression of TAZ and promote its nuclear translocation during the experiment. In the meantime, ChIP experiment confirmed that TAZ was a direct target molecule for BMP9/Smad signaling pathway. Following, we studied the effects of TAZ in osteogenic differentiation of MSCs and MMCs induced by BMP9 with using the ALP activity, ALP staining, calcium deposition, expression of osteogenic-related factors, and subcutaneous ectopic osteogenesis. Our results demonstrated that TAZ significantly promoted the early and late osteogenic differentiation induced by BMP9. Moreover, in vivo experiments showed that TAZ promoted the subcutaneous ectopic osteogenesis in nude mice. Mechanically, TAZ increased the levels of phosphorylation of p38, ERK1/2, JNK, and Smad1/5/8, and the total protein level of β-catenin in MSCs and MMCs. Therefore, our results showed that TAZ could promote osteogenic differentiation of BMP9-induced MSCs and MMCs in vitro and in vivo. Briefly, the effect of TAZ on the osteogenic differentiation of BMP9-induced MSCs can be summarized in Fig. 7.

There are a variety of indicators during the process of osteogenic differentiation and bone mineralization, of which RUNX2 is particularly important. RUNX2 is indispensable for the process of intramembranous ossification and osteoblast differentiation, which regulates the expression of osteogenesis-related genes such as osteocalcin, osteopontin, and type I collagen [54]. Meanwhile, DLX5, a vital indicator that reflected the late stage of osteogenic differentiation, promotes osteogenic differentiation by enhancing the expression of RUNX2 [55, 56]. Through the RT-PCR and western blot, we have found that TAZ would promote the expression of osteogenic differentiation indicators RUNX2, OCN, OPN, and DLX5 induced by BMP9. While we also found that, the silence TAZ would inhibit the expression of osteogenic genes. Previous studies have showed that the osteogenic differentiation was promoted by BMP9 through BMPs/Smad signaling pathway [32, 33]. When BMP9 was binding to the BMP/TGFβ-β signaling pathway receptors, Smad1/5/8 is phosphorylated. Then, the phosphorylated Smad1/5/8 combines with Smad4 to form a complex, which will promote the transcription of downstream genes [31]. Besides, TAZ regulated transcriptional regulation by binding to intranuclear Smad [57]. Hence, we believe that TAZ and BMP9 have synergistic effects in the classical Smad1/5/8 signaling pathway, which will promote the osteogenic differentiation of MSCs and MMCs better than BMP9 alone. In this study, we found that TAZ could increase the BMP9-induced the phosphorylation level of Samd1/5/8 without affecting total Samd1/5/8. Nevertheless, the phosphorylation level of Samd1/5/8 would be inhibited by silence TAZ. Consequently, the BMP9-induced osteogenic differentiation of MSCs and MMCs was enhanced by TAZ with activating classical Smad1/5/8 signaling pathway.

Simultaneously, the classical Wnt/β-catenin signaling pathway is also involved in the osteogenic differentiation of MSCs induced by BMP9 [45]. The Wnt/β-catenin signaling pathway is a highly conservative signaling pathway in evolution, and plays a significant regulatory role in embryonic development, cell appreciation, differentiation, and stem cell self-renewal [58]. The classical Wnt/β-catenin pathway is activated when the ligand Wnt binds to the receptor Frizzled (Fz) and low-density lipoprotein receptor-associated protein 5/6 (LRP5/6) on the cell membrane. Then β-catenin enters the nucleus and binds to the transcription factor TCF/LEF that to regulate transcription of downstream target genes. However, in the absence of Wnt, the cytoplasmic β-catenin binds to the destructive complex formed by the scaffold proteins APC, Axin2, the related protein kinases GSK3 and CK1α, and then β-catenin is degraded by ubiquitination which led to the inactivation of Wnt signaling pathway [58–61]. By western blot we have found that TAZ increased total β-catenin protein level in MSCs and MMCs induced by BMP9; while the total β-catenin protein level was inhibited by the silence TAZ. These results remind us that Wnt/β-catenin signal pathway may play a key regulatory role in TAZ by promoting the osteogenic differentiation of BMP9-induced MSCs and MMCs.

Apart from the classical Smad1/5/8 signaling pathway and Wnt/β-catenin signaling pathway, BMP9 promoted the osteogenic differentiation of MSCs by activating the MAPKs signaling pathways [62–64]. MAPK protein kinases, including p38, JNK, and ERK1/2, are mainly involved in the regulation of embryonic development, cell differentiation, proliferation, and death [65]. Our previous study had confirmed that p38/MAPK signaling pathway is also involved in the COX2-induced osteogenic differentiation of MSCs induced by BMP9 [46]. Furthermore, the osteogenic differentiation of mouse embryonic fibroblasts in the MAPKs-dependent manner was co-regulated by the CCN3 and DLL1 [47]. In our research, compared to treatment with BMP9 alone, the phosphorylation level of p38, JNK, and ERK1/2 was increased by the combination of TAZ and BMP9. However, the phosphorylation level of p38, JNK, and ERK1/2 was inhibited by the silence TAZ. Our results strongly suggested that the osteogenic differentiation of MSCs and MMCs of BMP9-induced was promoted by TAZ by activating MAPKs (p38, JNK, and ERK) signaling pathways.

In conclusion, we have sufficient evidence in this study that TAZ would promote the osteogenic differentiation of MSCs and MMCs induced by BMP9 through Samd1/5/8, Wnt/β-catenin, and MAPKs signal pathways. This trial extends our understanding of the biological function of BMP9 and has great significance for the early clinical application of BMP9.

## Materials and methods

### Cell line and reagents

HEK293 (human embryonic kidney 293 cells), C3H10T1/2 (murine mesenchymal stem cell line), MEFs (murine embryonic fibroblast), C2C12 (murine myoblasts) (obtained from the American Type Culture Collection, ATCC). Cells were cultured in Dulbecco’s modified eagle medium (DMEM, Hyclone) supplemented with 100 μg/mL of penicillin and 100 U/mL streptomycin (Hyclone, USA), 10% fetal bovine serum (Excell Bio, China) and incubated at 37 °C with 5% CO_2_.

Anti-phospho-Smad1/5/8 (#D5B10), anti-ERK1/2 (#4695), anti-phospho-ERK1/2 (#4370), anti-p38 (#9212), anti-phospho-p38 (#4511 S), anti-JNK (#9252), anti-phospho-JNK (#9255), and anti-β-catenin (#8480) antibodies (obtained from Cell Signaling Technology, Danvers, MA, USA); Anti-osteocalcin (DF12303), anti-Smad1/5/8 (AF0614) antibodies from Affinity Biosciences, China; anti-osteopontin (A5427) and anti-RUNX2 (A5193) antibodies from Bimake; anti-β-actin (#TA-09), from Zhongshan Golden Bridge Biotechnology, Beijing, China; verteporfin (TAZ inhibitor) from Selleck (Shanghai, China); TM-25659 (TAZ agonist) from MedChemExpress (MCE).

### Construction of recombinant adenoviruses

The recombinant adenoviruses Ad-TAZ and Ad-si-TAZ were constructed by the Ad-easy system [66]. Ad-GFP and Ad-RFP were used for tracking tags. The Ad-GFP, Ad-RFP, and Ad-BMP9 were presented by Professor Tong-Chuan He of University of Chicago Medical Center (Chicago, IL, USA).

### Alkaline phosphatase activity and staining

Cells were inoculated in 24-well plates, and treated by various treatment in accordance with the experiment design. Briefly, for Ad-TAZ: cells were infected with Ad-TAZ and/or Ad-BMP9. For Ad-si-TAZ: cells were infected with Ad-si-TAZ and/or Ad-BMP9. For TM-25659 and Verteporfin: cells were infected with Ad-BMP9 and treated with various concentrations of TM-25659 (5, 10, 15, and 20 μM) or Verteporfin (0.2, 0.4, 0.6, and 0.8 μM). And cells were cultured in preliminary medium. ALP staining was detected using an ALP assay kit (Solarbio, Beijing, China) for 5 and 7 days after treatment following the reagent instructions. Alkaline phosphatase activity: Cells were lysed with 1× cell lysis buffer (200 μL/well) for 5 min in the dark, and centrifuged. Five microliters of cell lysate supernatant were aspirated into ALP working solution (5 μL ALP substrate plus +15 μL buffer) for 45–60 min in the dark and tested on a Hitachi 7060 C automatic biochemical analyzer. Each experiment was repeated three times independently.

### Alizarin red S staining

Cells were inoculated in 24-well plates, and treated by various treatment in accordance with the experiment design. Shortly, for Ad-TAZ: cells were infected with Ad-TAZ and/or Ad-BMP9. For Ad-si-TAZ: cells were infected with Ad-si-TAZ and/or Ad-BMP9. For TM-25659 and Verteporfin: cells were infected with Ad-BMP9 and treated with various concentrations of TM-25659 (5, 10, 15, and 20 μM) or Verteporfin (0.2, 0.4, 0.6, and 0.8 μM). Then cells were cultured in 10% calcium salt medium (containing 50 mg/L ascorbic acid and 10 mmol/L β-glycerophosphate disodium salt). Alizarin Red S Staining was performed for 14 and 21 days after treatment, as previously described [31]. Briefly, cells were fixed with 4% paraformaldehyde for 10–20 min, washed for three times with PBS solution, and extensively washed with PBS solution after the 0.05% alizarin red dye solution (200 μL/well) was added. The results were recorded under a microscope and the images were saved after scanning. Each experiment was repeated three times independently.

### Total RNA and quantitative real-time PCR (qRT-PCR)

Total RNA was extracted with Trizol reagents (Takara, Otsu, Japan) and reverse transcription was executed by using the Takara Prime Script RT Reagent Kit. Quantitative real-time PCR (qRT-PCR): the first-strand cDNA products were diluted 25-fold for qRT-PCR detection in a system of 2.5 ul upstream and downstream primers, 2.5 ul ddH_2_O, 2.5 ul SYBR Green and 2.5 ul cDNA. Reaction condition: 95 °C for 3 min, 95 °C for 20 s, 66 °C for 10 s, 3 cycles; 95 °C for 20 s, 55 °C for 10 s and 70 °C for 1 s, 36 cycles, and fluorescence was collected at the end of extension. 65–95 °C dissolution analysis. All samples were normalized according to the expression level of GAPDH. Each experiment was repeated three times independently. All primers used are presented in Table 1.

**Table 1:** Primer sequence.

Gene name	Forward primer	Reverse primer
GAPDH	GGCTGCCCAGAACATCAT	CGGACACATTGGGGGTAG
OPN	ACACTTTCACTCCAATCGTCC	TGCCCTTTCCGTTGTTGTCC
OCN	TCTGACAAAGCCTTCATGTCC	AAATAGTGATACCGTAGATGCG
RUNX2	GGTGAAACTCTTGCCTCGTC	AGTCCCAACTTCCTGTGCT
DLX5	CTCAGCCACCACCCTCAT	TGGCAGGTGGGAATTGAT
OSX	GGGAGCAGAGTGCCAAGA	TACTCCTGGCGCATAGGG
ID1	ACGACATGAACGGCTGCT	CAGCTGCAGGTCCCTGAT
ID2	CAGCATCCCCCAGAACAA	TCTGGTGATGCAGGCTGA
ID3	CTACGAGGCGGTGTGCTG	GCGCGAGTAGCAGTGGTT

### Nucleoprotein extraction

Cells were inoculated in cell culture dishes, and treated by various treatments according to the experiment design for 24 h. Shortly, cells were infected with Ad-BMP9 or Ad-GFP. The nucleoprotein was extracted by using Nuclear Protein Extraction Kit (Solarbio, Beijing, China) according to the manufacturer’s recommendations.

### Western blot

Cells were lysed with lysis buffer (Beyotime, Haimen, China) for total protein. The protein concentration was determined by BCA assay. The protein was separated by SDS-PAGE, and then transferred to a PVDF membrane. After blocking with bovine serum albumin (BSA) for 2 h, the membrane was incubated with the corresponding primary antibody. The membrane was washed with TBST for three times, followed by incubation with a secondary antibody conjugated with horseradish peroxidase at 37 °C for 1 h. Protein of interest was visualized by an ECL kit, and photographed by a chemiluminescence imaging system. The quantification of western blot was performed by Image Lab software. Each experiment was repeated three times independently.

### Ectopic bone formation model

Cells were inoculated in cell culture dishes, and treated by various treatments in accordance with the experiment design. Briefly, the cells were infected with Ad-BMP9 and Ad-TAZ or Ad-si-TAZ. After treatment for 24 h, the cells were collected for subcutaneous injection (5 × 10^6^ cells per injection) into the flanks of athymic nude mice (Randomly assigned to one of four group, three animals per group, 4–6-week-old male, the nude mice were purchased from Beijing Huafukang Biotechnology Co., Ltd). Four weeks after injection, the animals were euthanized and bone blocks were collected for micro-CT imaging and histological evaluation. All animal experiments in this trial were approved by the ethics committee of Chongqing Medical University (The approval animal protocol number: 2022-K198). The housing facility of nude mouse is a barrier housing facility, and it has in keeping with national standard “Laboratory Animal—Requirements of Environment and Housing Facilities” (GB 14925–2010). The care of laboratory animal and the animal experimental operation have conforming to “Chongqing Management Approach of Laboratory Animal” (Chongqing government order NO.195).

### Chromatin Immunoprecipitation (ChIP) analysis

The MSCs were infected by Ad-GFP or Ad-BMP9. At 48 h after infection, cells were crosslinked and subjected to ChIP analysis using ChIP Assay Kit (Beyotime, Haimen, China) in accordance with the recommendations of manufacturers. Smad1/5/8 antibody (Proteintech Group, Inc) or control IgG was used to pull down the protein-DNA complexes. The presence of TAZ promoter sequence was detected by using four pairs of primers corresponding to mouse TAZ promoter region. Each experiment was repeated three times independently.

### Micro-CT imaging analysis, H&E, Alcian Blue, and Masson’s trichrome staining

Bone mass scanning and three-dimensional reconstruction were performed using a Micro-CT instrument, and quantitative analysis of osteogenesis was performed. Decalcification was performed on the experimental bone blocks, which were then embedded in paraffin. The bone blocks embedded in paraffin were sectioned, and then subjected to H&E staining, Mason staining and Alcian Blue staining. Each experiment was repeated three times independently.

### Statistical analysis

Results were analyzed by GraphPad Prism 5.0 (San Diego, CA, USA) and SPSS 18.0 (SPSS, Inc., Chicago, IL, USA) software package. Data from three independent experiments were displayed as mean ± standard deviation (SD), and statistical significance in groups was analyzed by one-way ANOVA. Multiple comparisons in groups were performed using Tukey’s post hoc test. *P* < 0.05 was considered statistically significant.

## Supplementary information

supplemental figure

the original western blots

## Data Availability

The corresponding author will provide the original data that used to support the findings of this study upon reasonable request.
