# Supplementary material for: TAZ promotes osteogenic differentiation of mesenchymal stem cells line C3H10T1/2, murine multi-lineage cells lines C2C12, and MEFs induced by BMP9
Source: Cell Death Discov. 2022 Dec 27;8:499. doi: 10.1038/s41420-022-01292-y (PMC9794779; doi:10.1038/s41420-022-01292-y)
Supplement: Supplementary file 1 — supplemental figure [file 41420_2022_1292_MOESM1_ESM.docx]

Figure S1：
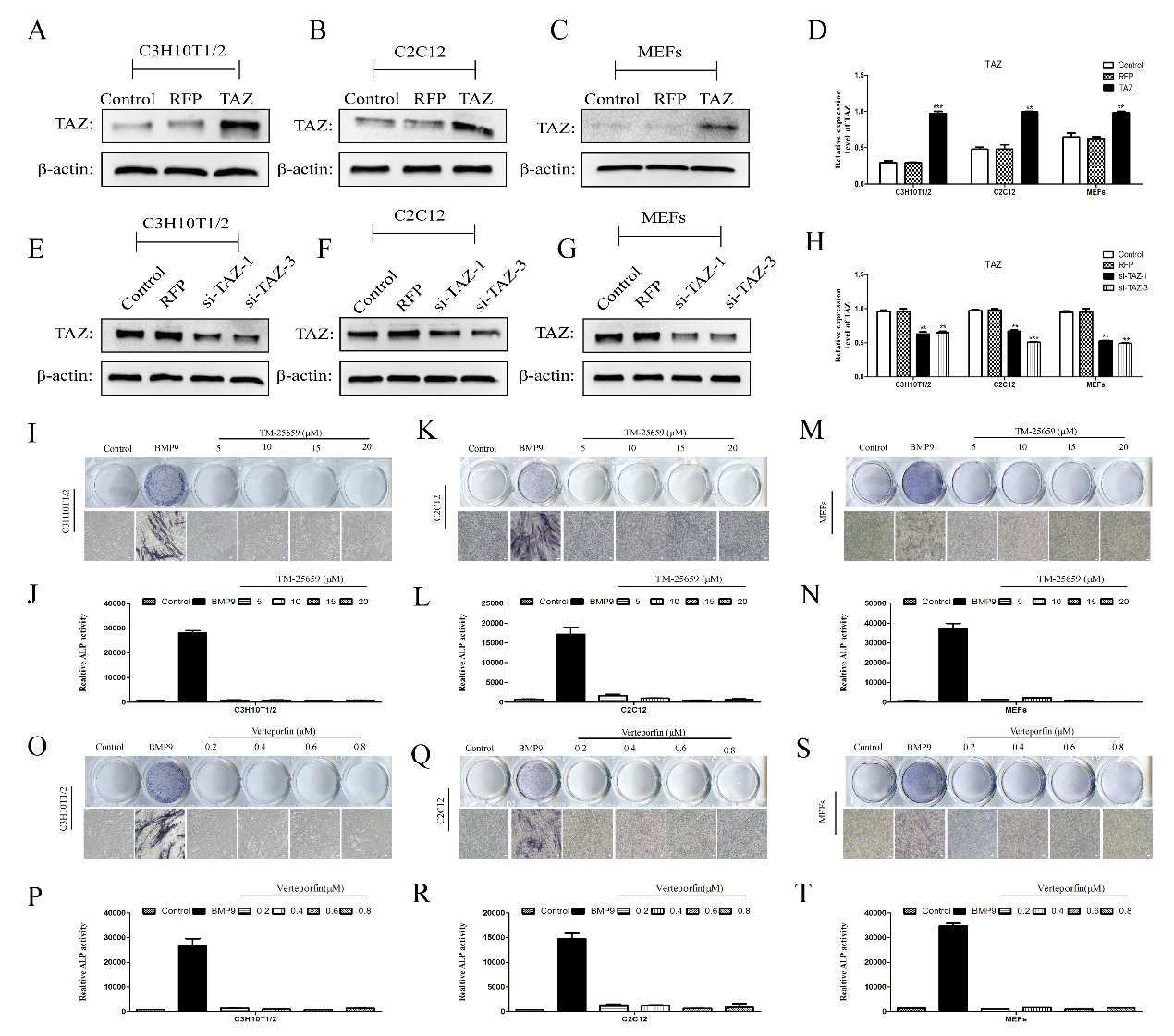


Figure S2：


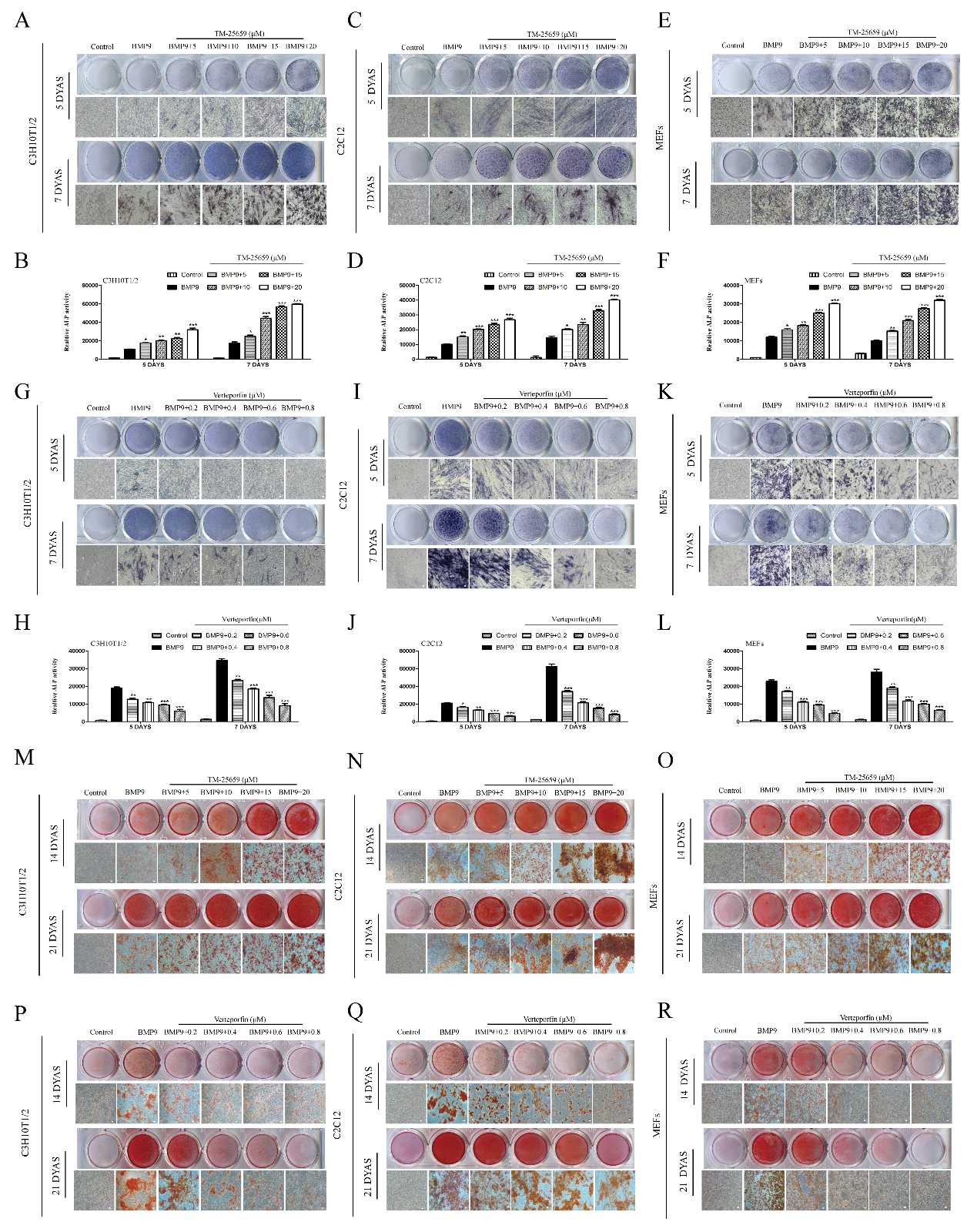


Figure S3：
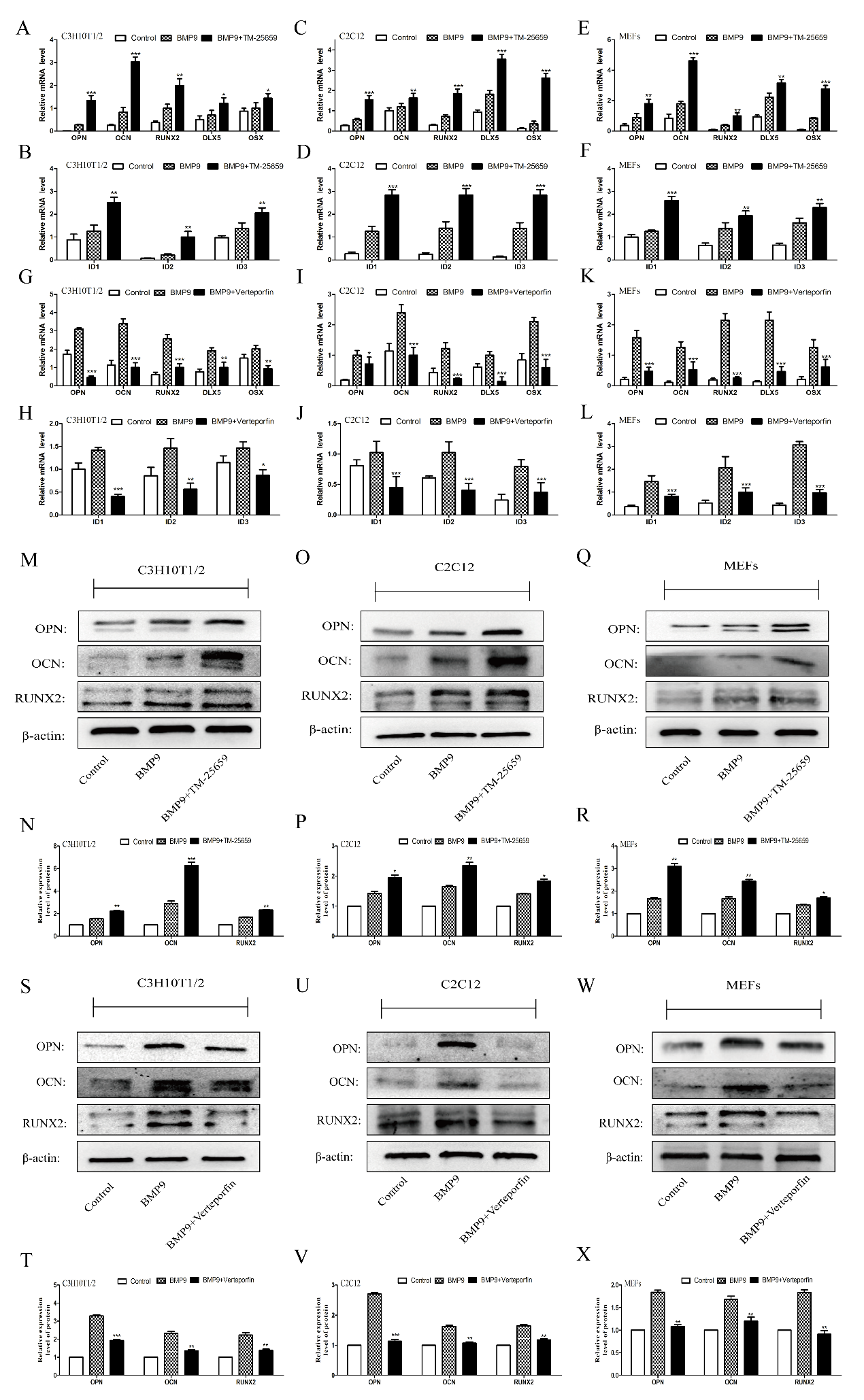
 Figure S4：
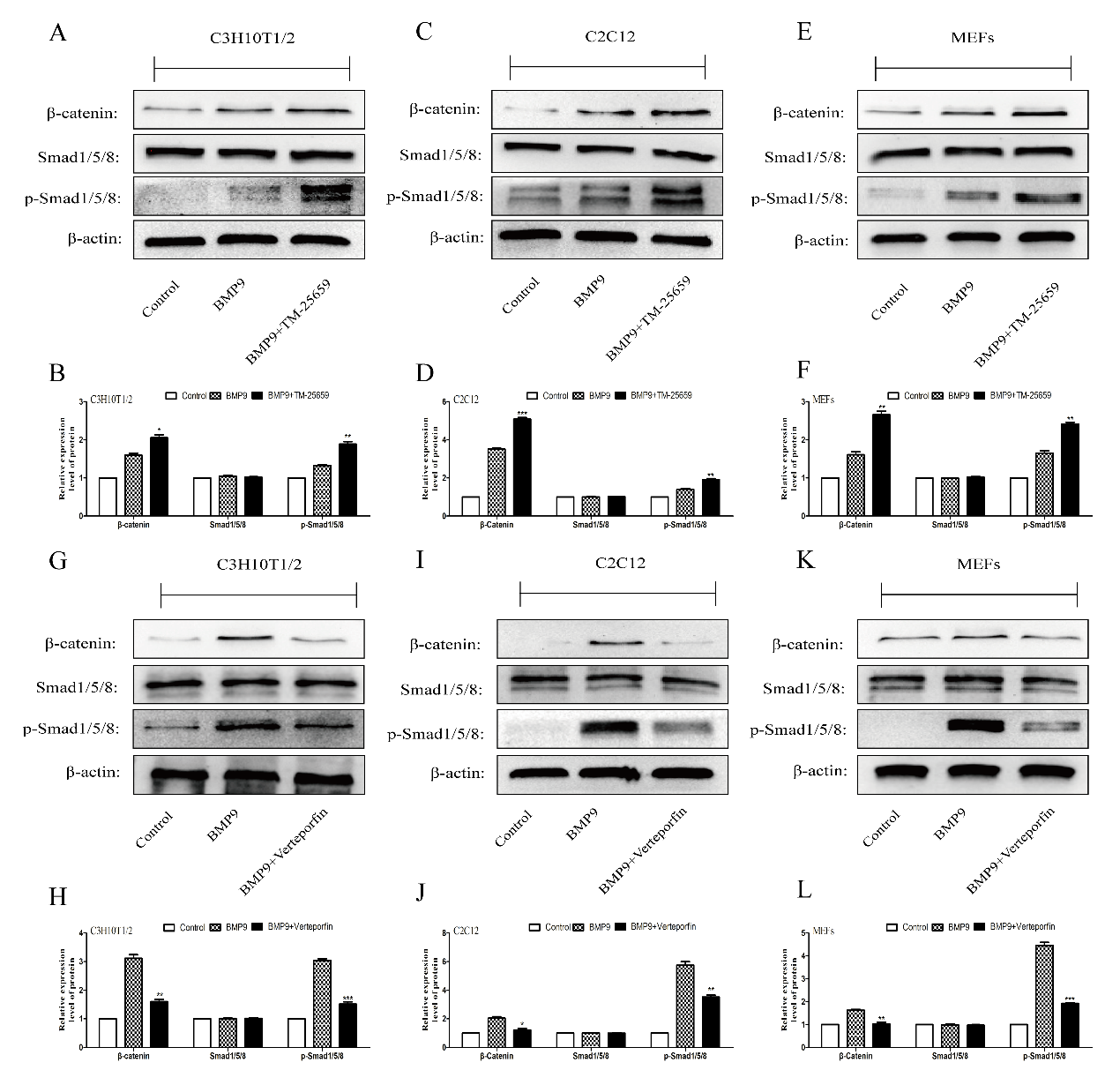


Figure S5：
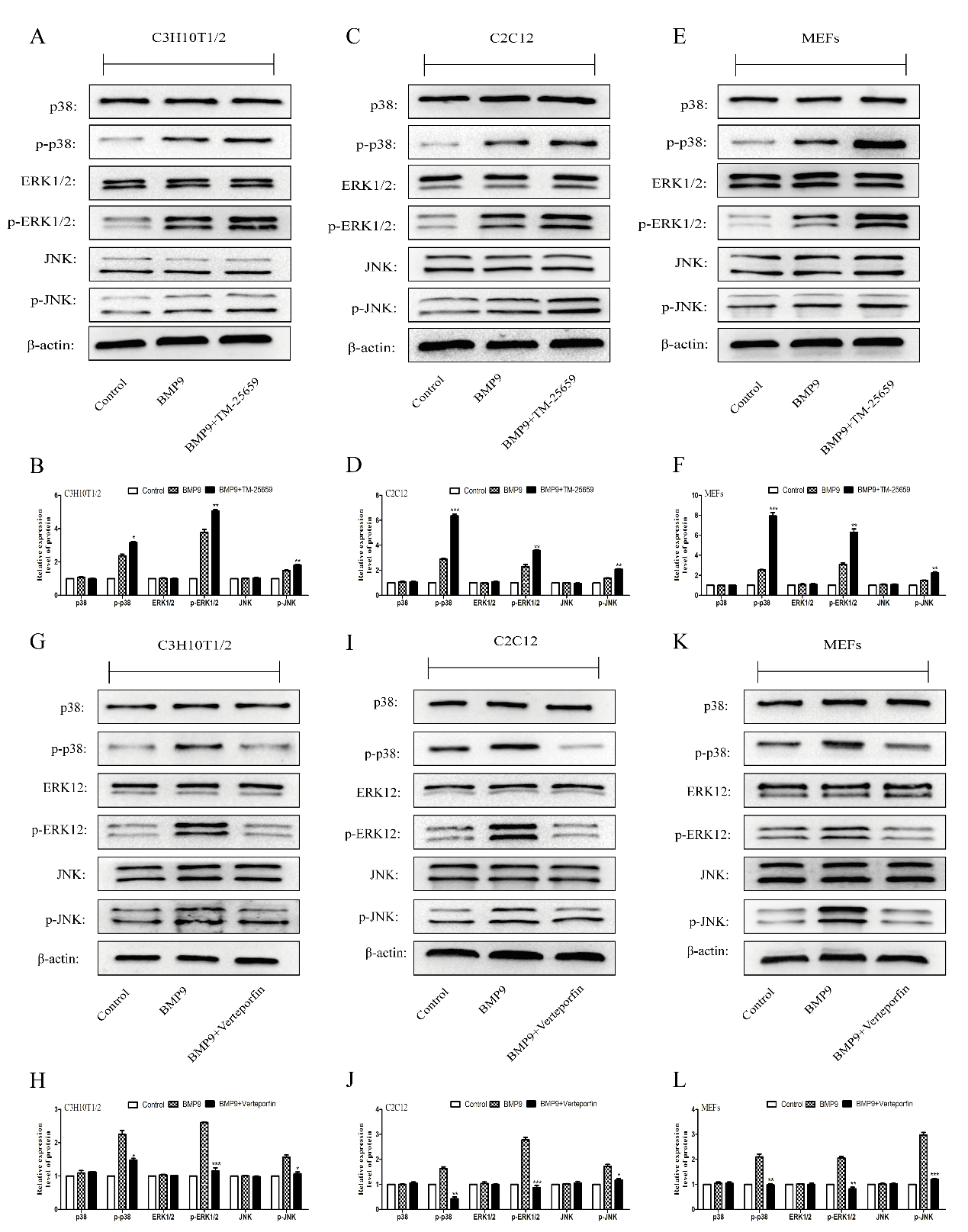


Figure S1：Validation of recombinant adenovirus Ad-TAZ and Ad-si-TAZ in MSCs and MMCs. (A-D) Cells were infected with Ad-TAZ, and the protein expression of TAZ was analyzed by Western blot. (E-H) Cells were infected with Ad-si-TAZ, and the protein expression of TAZ was analyzed by Western blot. (I-N) Cells were treated with BMP9 or TM-25659 (5, 10, 15, and 20 µM). The ALP activity was detected by chemiluminescence assay and ALP staining was detected by histochemical staining assay at 5 days. (O-T) Cells were treated with BMP9 or verteporfin (0.2, 0.4, 0.6, and 0.8 µM). The ALP activity was detected by chemiluminescence assay and ALP staining was detected by histochemical staining assay at 5 days. The data are shown as mean ± SD for three separate experiments. (*P<0.05, **P<0.01, ***P<0.001). Ad-RFP: Adenovirus carrying red fluorescent protein gene.

Figure S2：The effect of TAZ on BMP9-induced early and late osteogenic differentiation in MSCs and MMCs. Cells were treated with TM-25659 (5, 10, 15, and 20 µM) or verteporfin (0.2, 0.4, 0.6, and 0.8 µM), followed by treatment with BMP9. (A-L) The ALP activity was detected by chemiluminescence assay and ALP staining was detected by histochemical staining assay at 5 days and 7 days. (M-R) Calcium deposition was analyzed by Alizarin Red S staining at 14 days and 21 days. The data are shown as mean ± SD for three separate experiments.

Figure S3 The effect of TAZ on BMP9-induced expression levels of pivotal osteogenic markers in MSCs and MMCs. Cells were treated with TM-25659 (5, 10, 15, and 20 µM) or verteporfin (0.2, 0.4, 0.6, and 0.8 µM), followed by treatment with BMP9. (A-L) The mRNA expression of RUNX2, OPN, OCN, DLX5, ID1, ID2 and ID3 were determined by qPCR. (M-X) The protein expression of RUNX2, OPN and OCN were detected by Western blot. The data are shown as mean ± SD for three separate experiments. (*P<0.05, **P<0.01, ***P<0.001)

Figure S4：The effect of TAZ on BMP9-induced classical Smad1/5/8 and Wnt/β-catenin in MSCs and MMCs. Cells were treated with TM-25659 (5, 10, 15, and 20 µM) or verteporfin (0.2, 0.4, 0.6, and 0.8 µM), followed by treatment with BMP9. ((A-L) The total amount and phosphorylated forms of Smad1/5/8 were measured by Western blot. (A-L) The total amount of β-catenin was measured by Western blot. The data are shown as mean ± SD for three separate experiments. (*P<0.05, **P<0.01, ***P<0.001)

Figure S5: The effect of TAZ on BMP9-induced MAPKs in MSCs and MMCs. Cells were treated with TM-25659 (5, 10, 15, and 20 µM) or verteporfin (0.2, 0.4, 0.6, and 0.8 µM), followed by treatment with BMP9. (A-L) The total amount of p38, ERK1/2 and JNK and the phosphorylated forms of p38, ERK1/2 and JNK were measured by Western blot. The data are shown as mean ± SD for three separate experiments. (*P<0.05, **P<0.01, ***P<0.001)
