# Supplementary material for: TAZ promotes osteogenic differentiation of mesenchymal stem cells line C3H10T1/2, murine multi-lineage cells lines C2C12, and MEFs induced by BMP9
Source: Cell Death Discov. 2022 Dec 27;8:499. doi: 10.1038/s41420-022-01292-y (PMC9794779; doi:10.1038/s41420-022-01292-y)
Supplement: Supplementary file 2 — the original western blots [file 41420_2022_1292_MOESM2_ESM.pptx]

## Slide 1
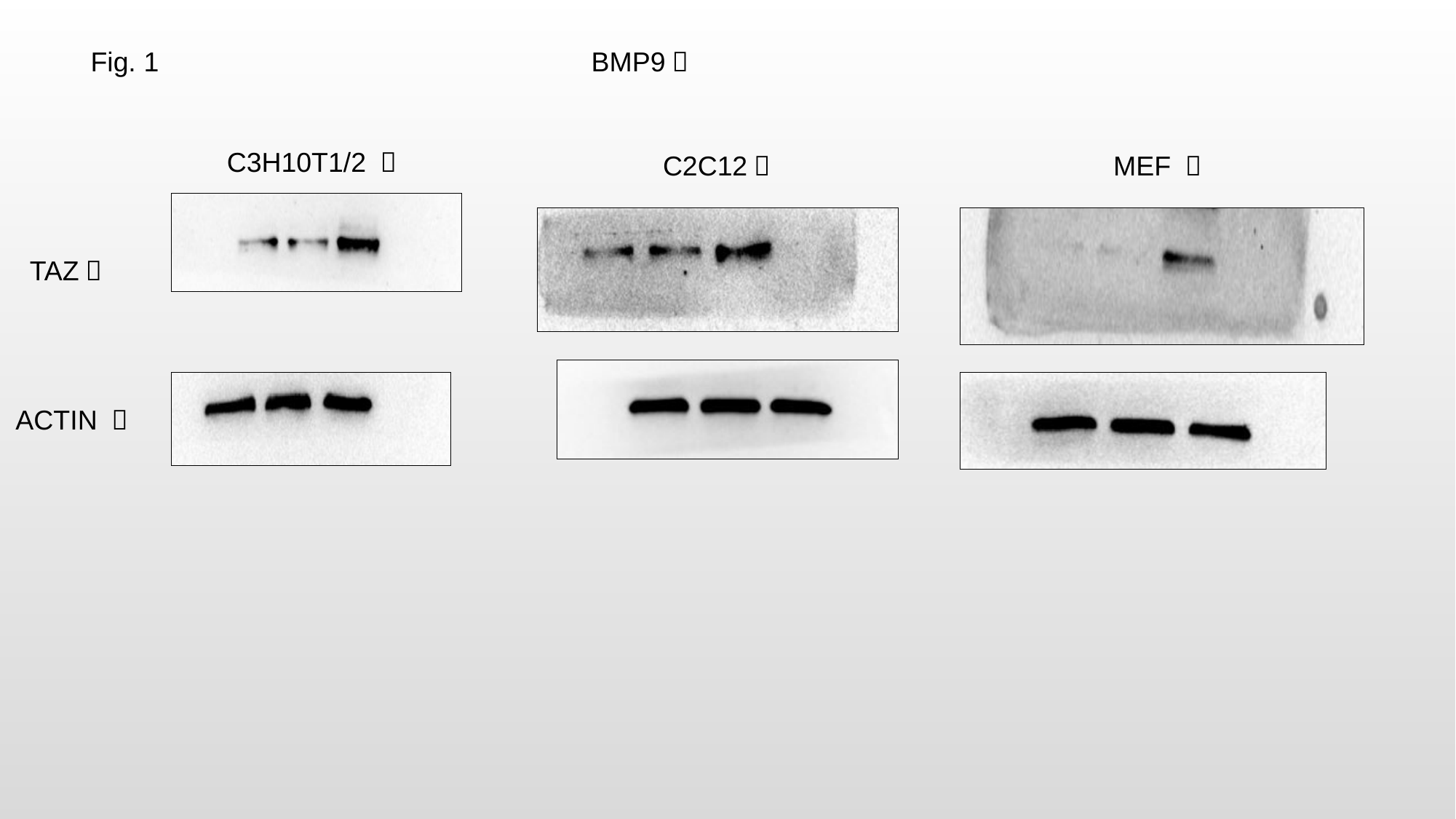

Fig. 1
BMP9：
C3H10T1/2 ：
C2C12：
MEF ：
TAZ：
ACTIN ：

## Slide 2
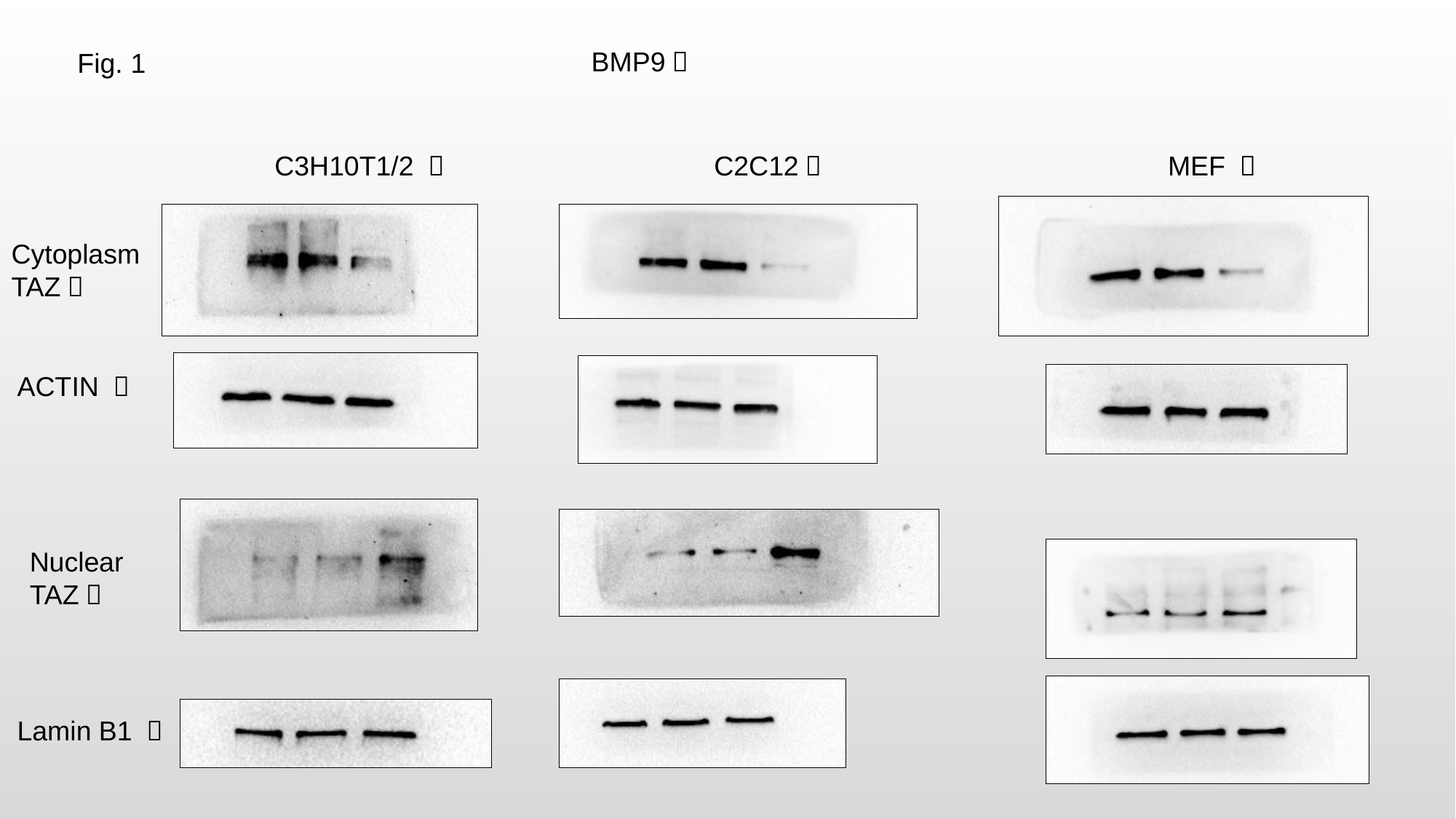

BMP9：
Fig. 1
C3H10T1/2 ：
C2C12：
MEF ：
CytoplasmTAZ：
ACTIN ：
NuclearTAZ：
Lamin B1 ：

## Slide 3
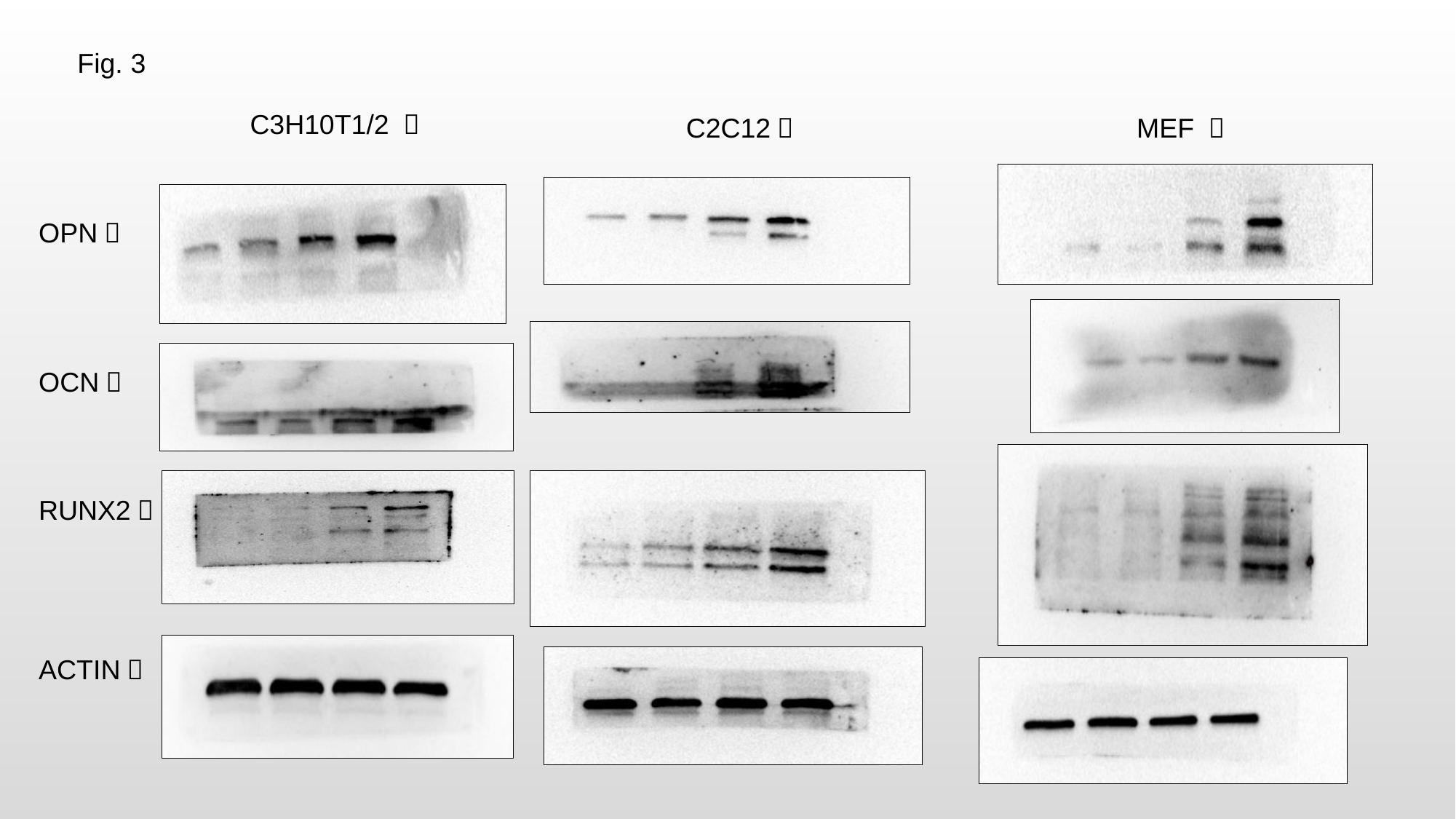

Fig. 3
C3H10T1/2 ：
C2C12：
MEF ：
OPN：
OCN：
RUNX2：
ACTIN：

## Slide 4
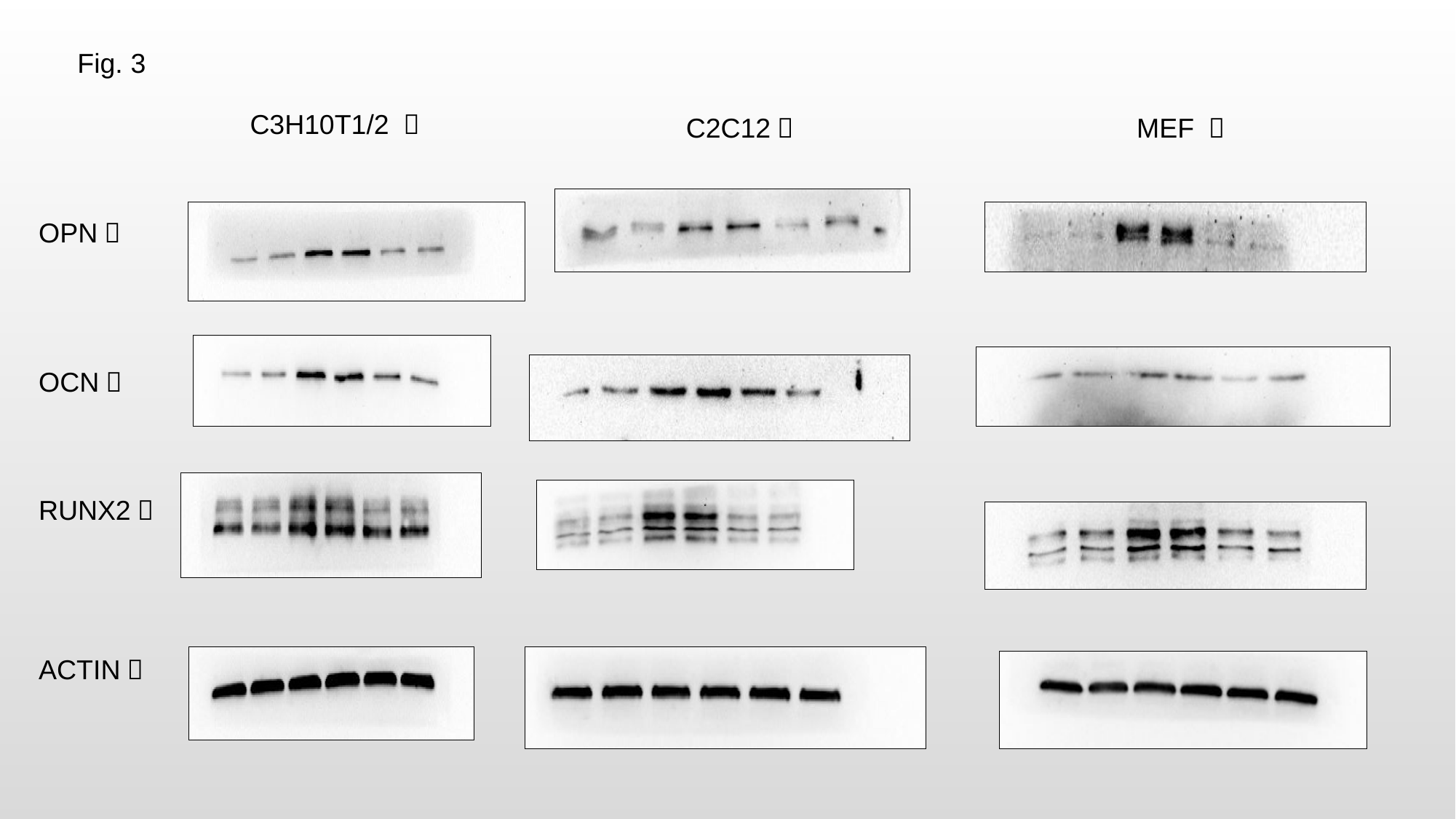

Fig. 3
C3H10T1/2 ：
C2C12：
MEF ：
OPN：
OCN：
RUNX2：
ACTIN：

## Slide 5
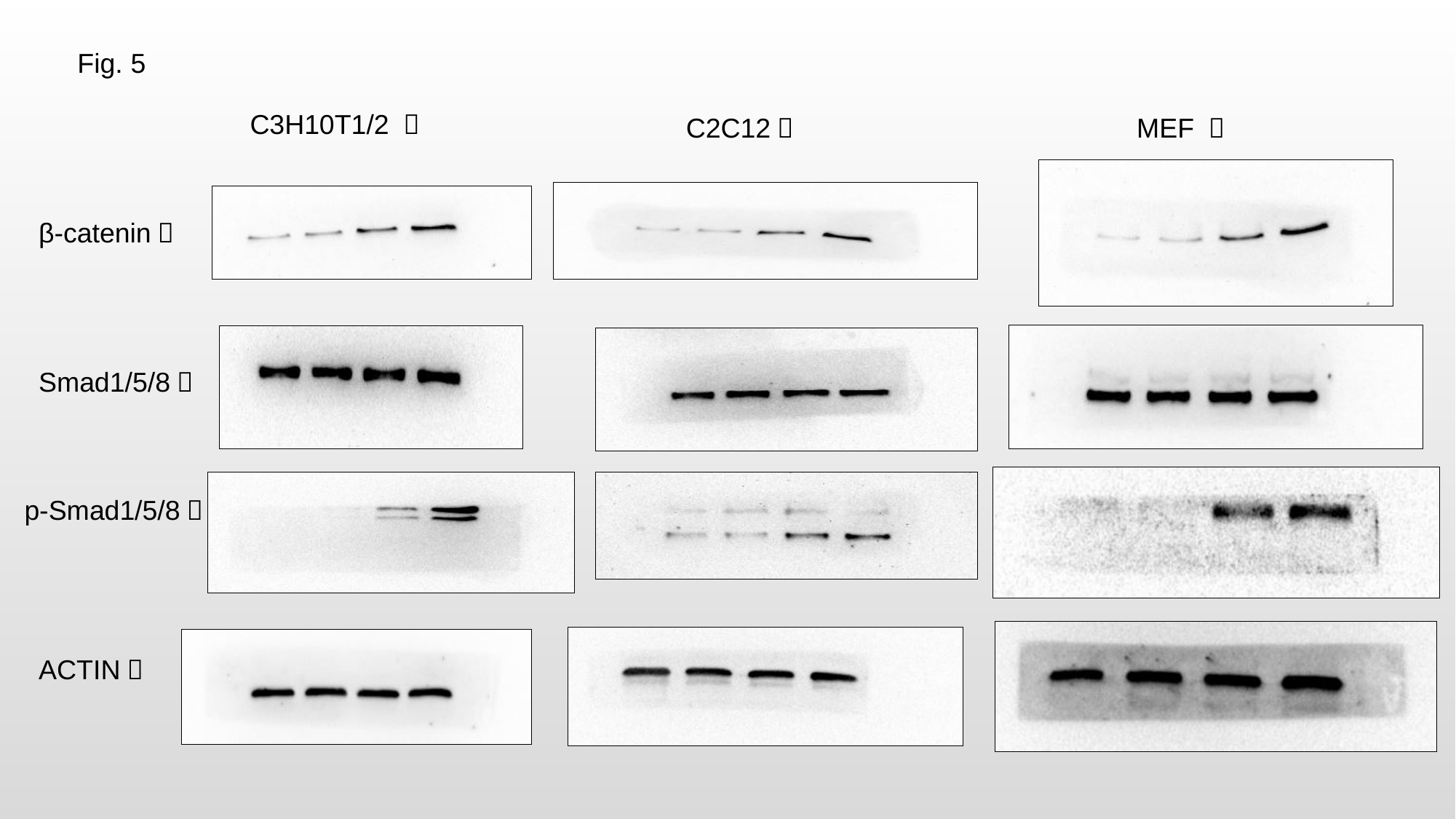

Fig. 5
C3H10T1/2 ：
C2C12：
MEF ：
β-catenin：
Smad1/5/8：
p-Smad1/5/8：
ACTIN：

## Slide 6
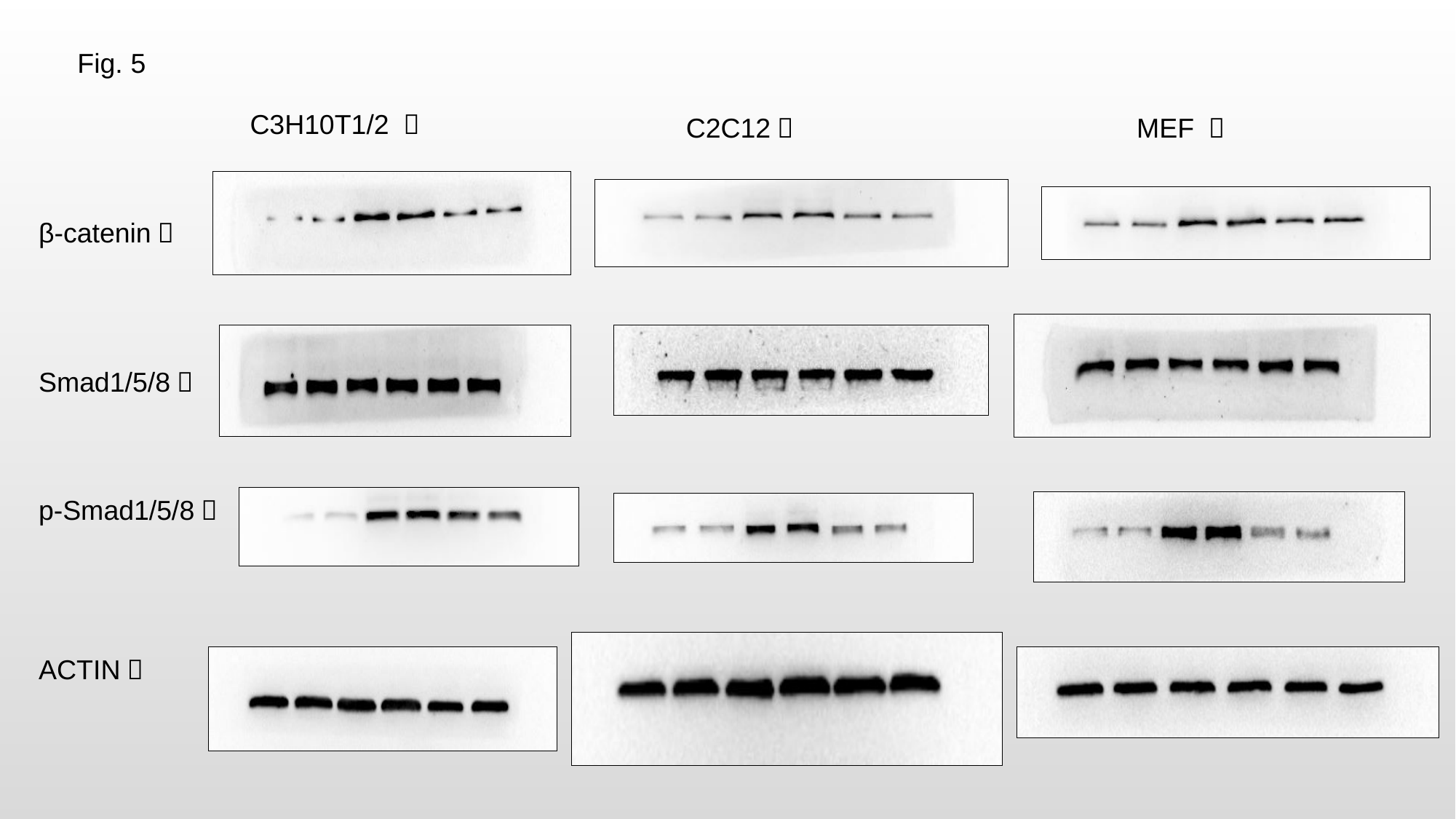

Fig. 5
C3H10T1/2 ：
C2C12：
MEF ：
β-catenin：
Smad1/5/8：
p-Smad1/5/8：
ACTIN：

## Slide 7
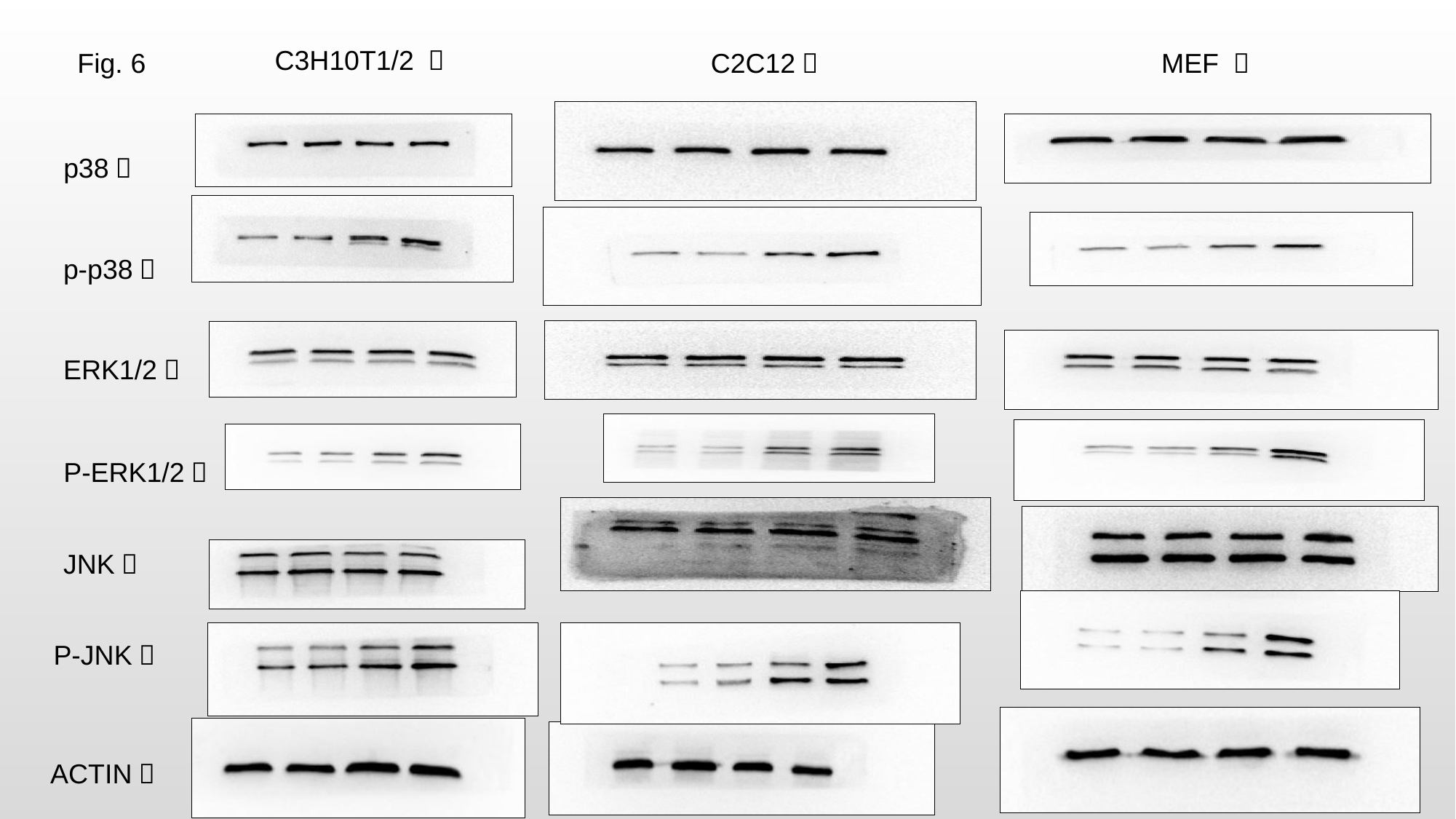

C3H10T1/2 ：
Fig. 6
C2C12：
MEF ：
p38：
p-p38：
ERK1/2：
P-ERK1/2：
JNK：
P-JNK：
ACTIN：

## Slide 8
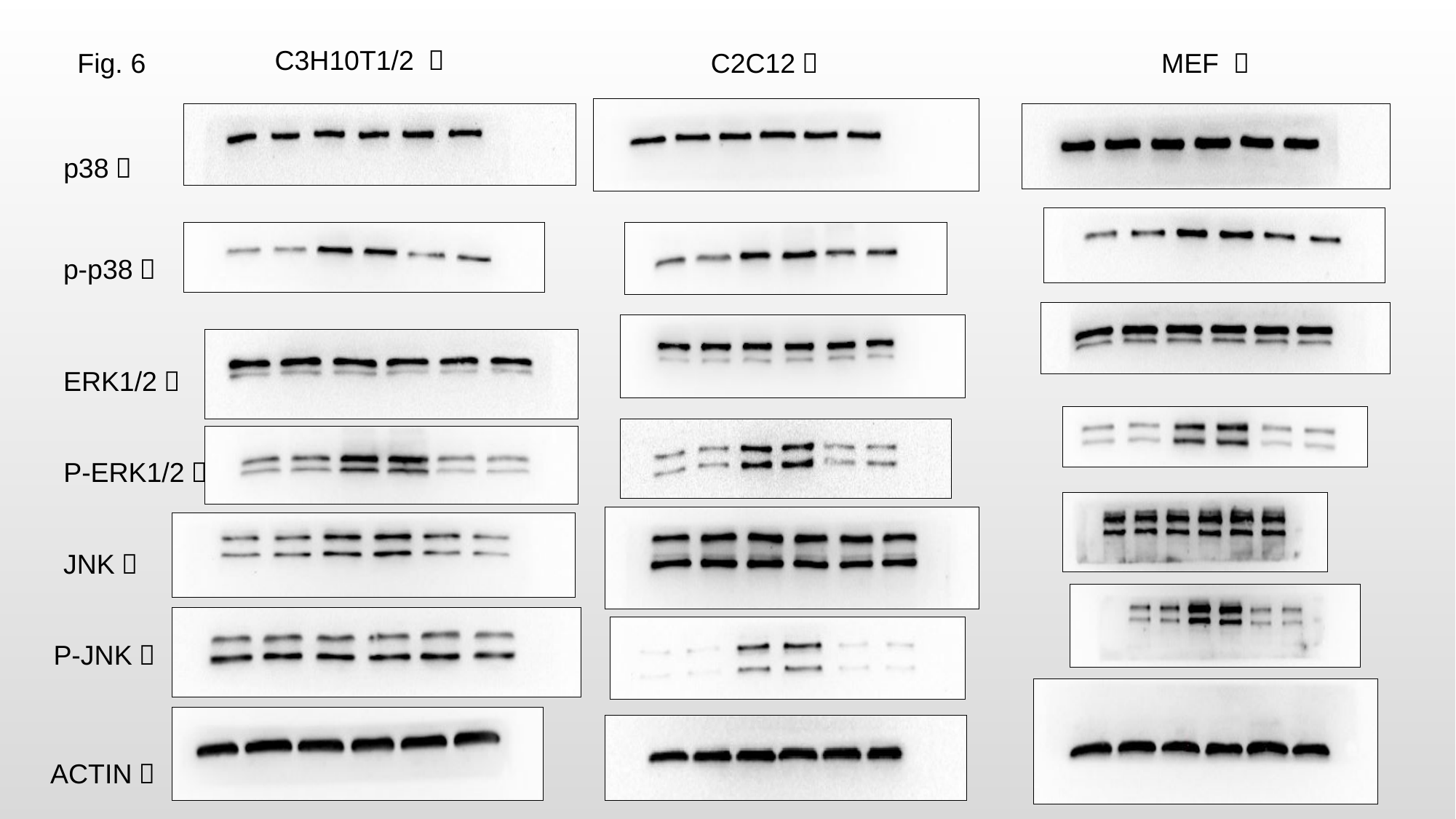

C3H10T1/2 ：
Fig. 6
C2C12：
MEF ：
p38：
p-p38：
ERK1/2：
P-ERK1/2：
JNK：
P-JNK：
ACTIN：

## Slide 9
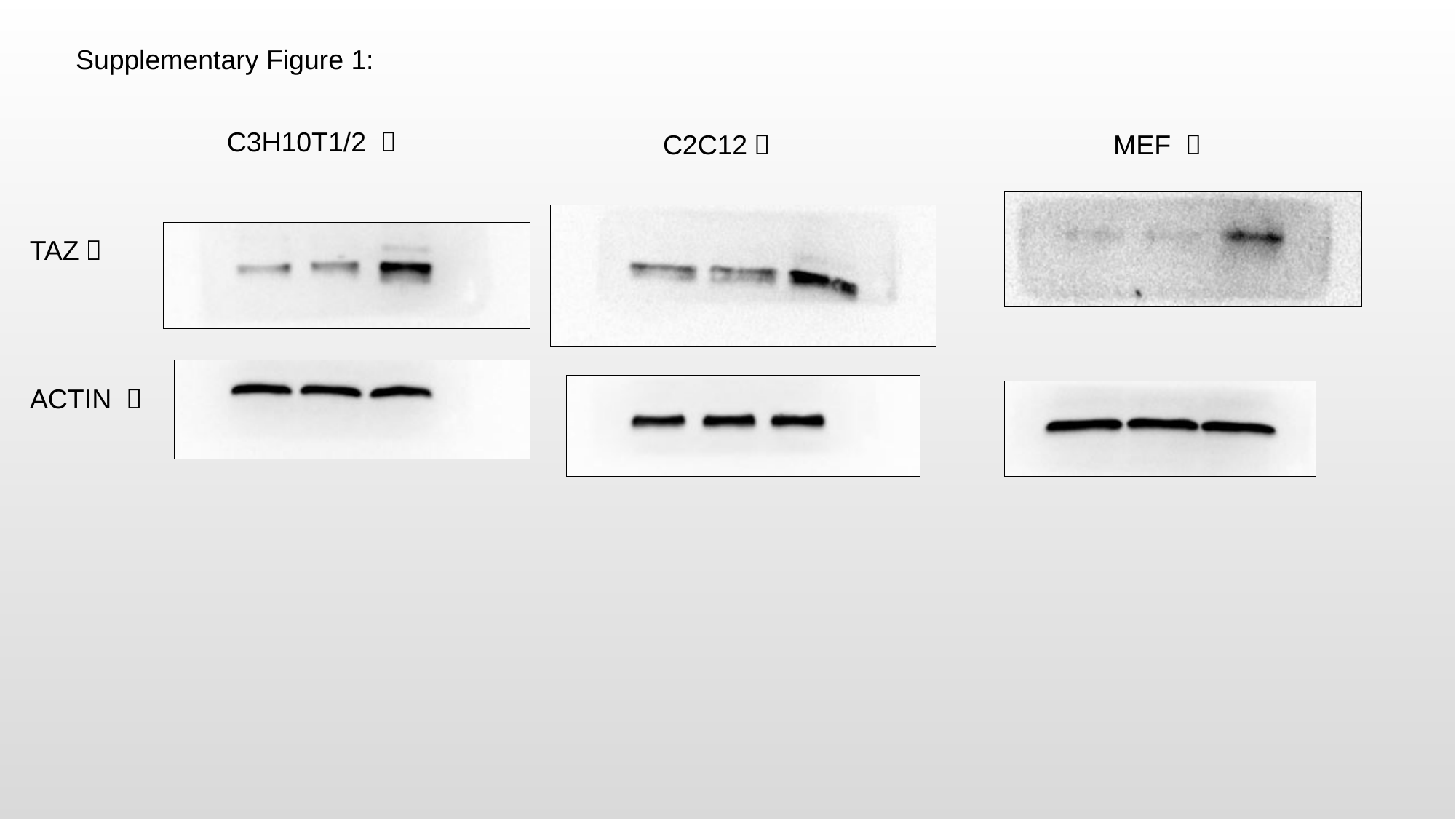

Supplementary Figure 1:
C3H10T1/2 ：
C2C12：
MEF ：
TAZ：
ACTIN ：

## Slide 10
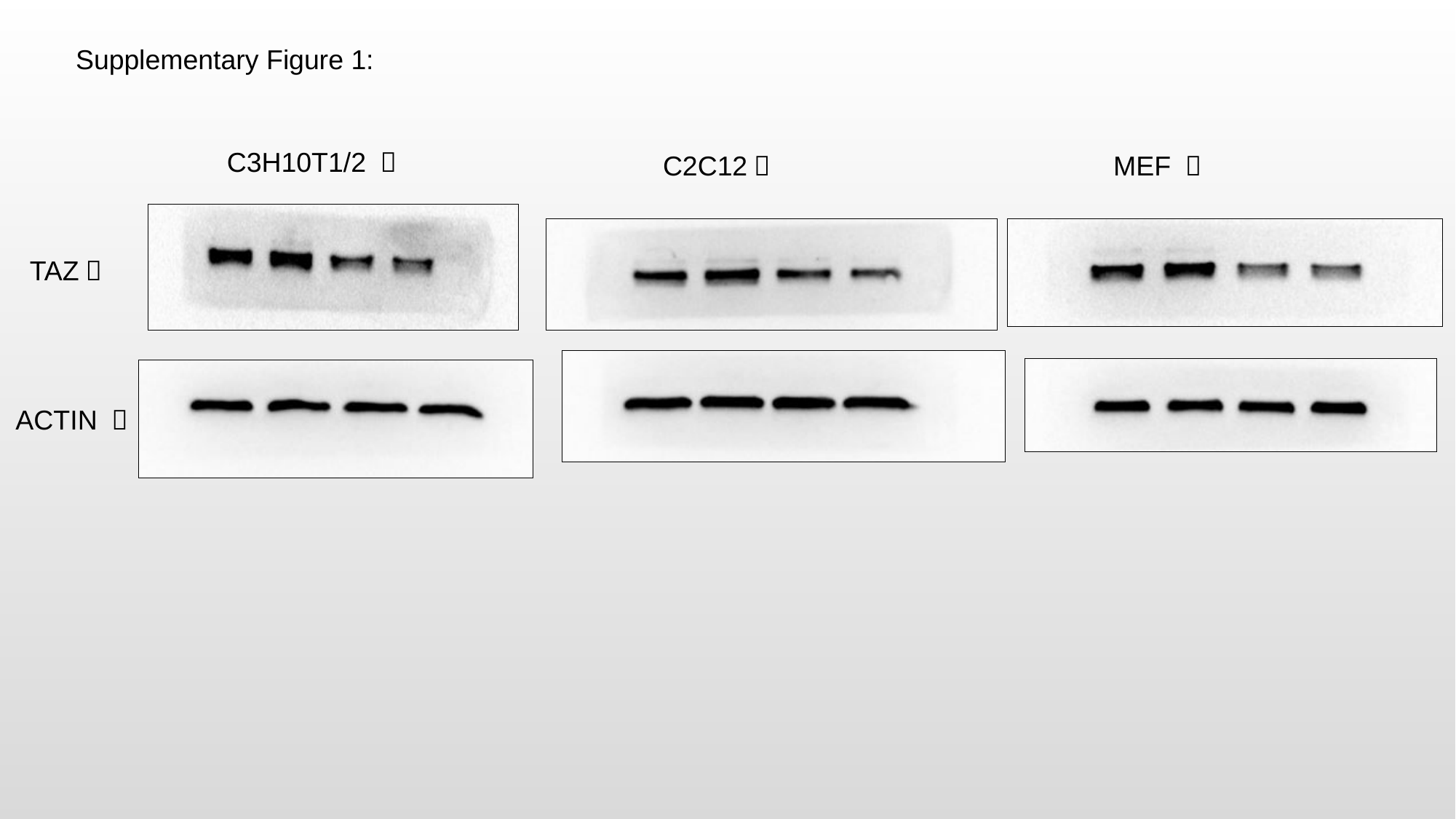

Supplementary Figure 1:
C3H10T1/2 ：
C2C12：
MEF ：
TAZ：
ACTIN ：

## Slide 11
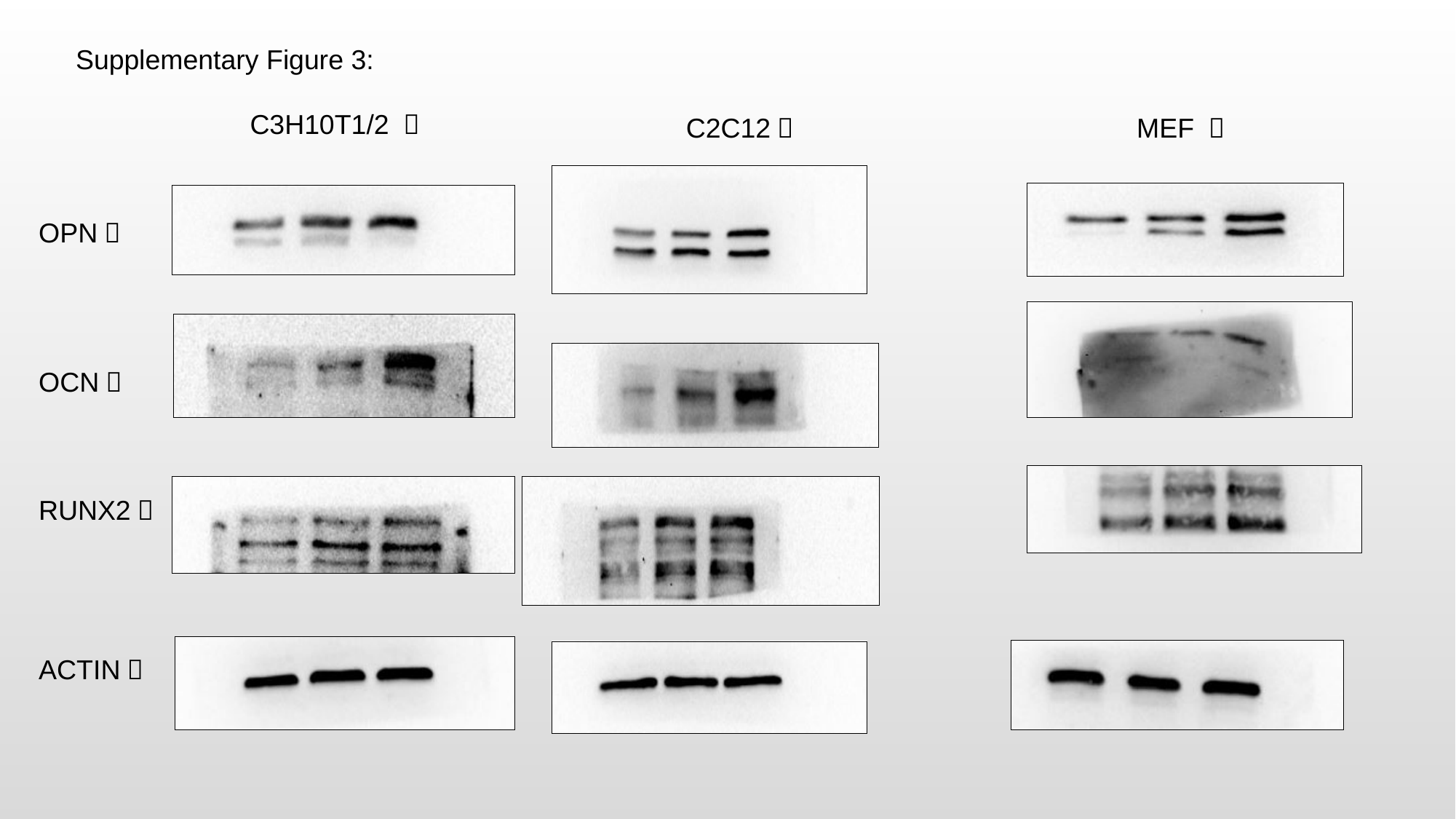

Supplementary Figure 3:
C3H10T1/2 ：
C2C12：
MEF ：
OPN：
OCN：
RUNX2：
ACTIN：

## Slide 12
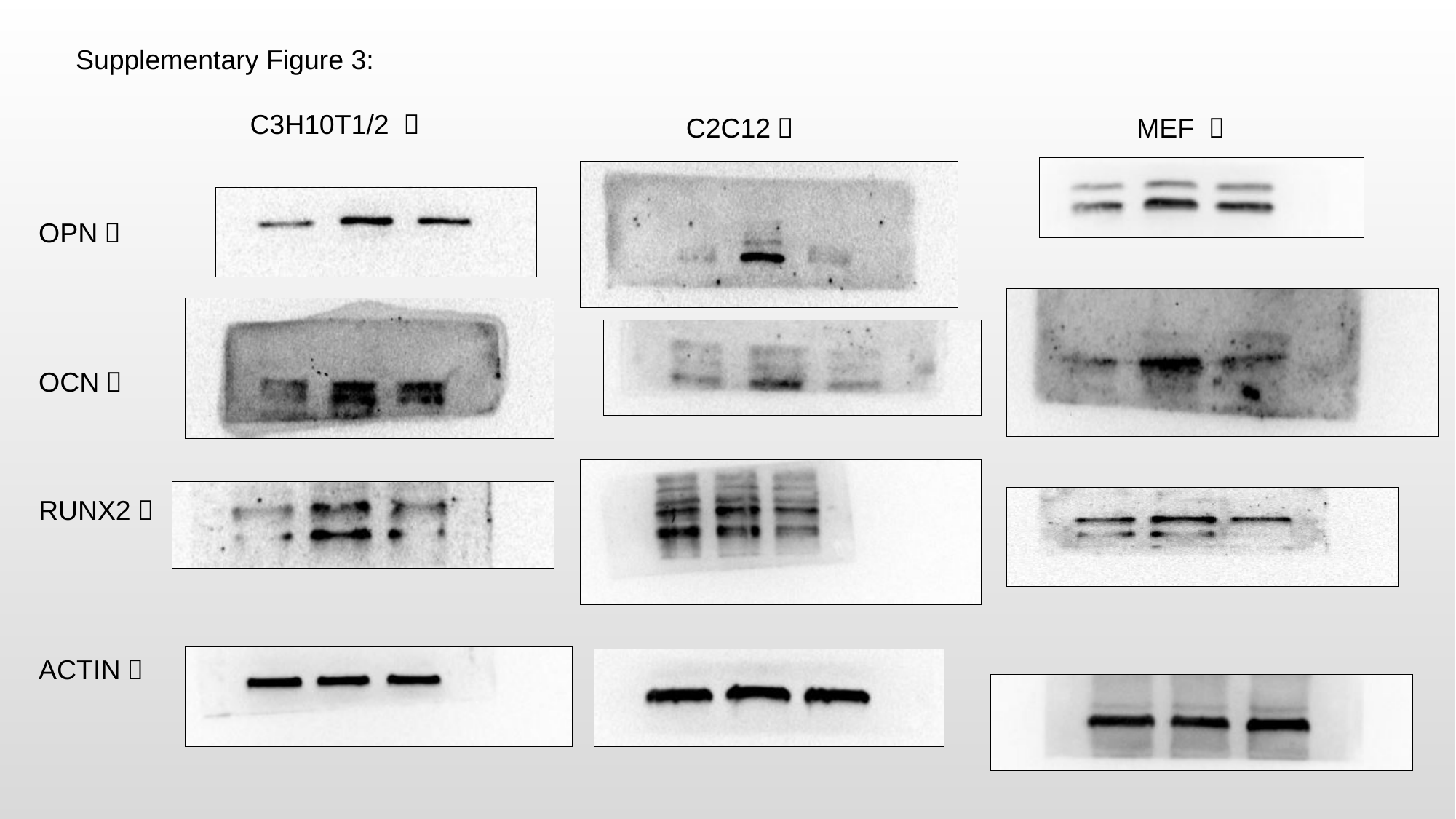

Supplementary Figure 3:
C3H10T1/2 ：
C2C12：
MEF ：
OPN：
OCN：
RUNX2：
ACTIN：

## Slide 13
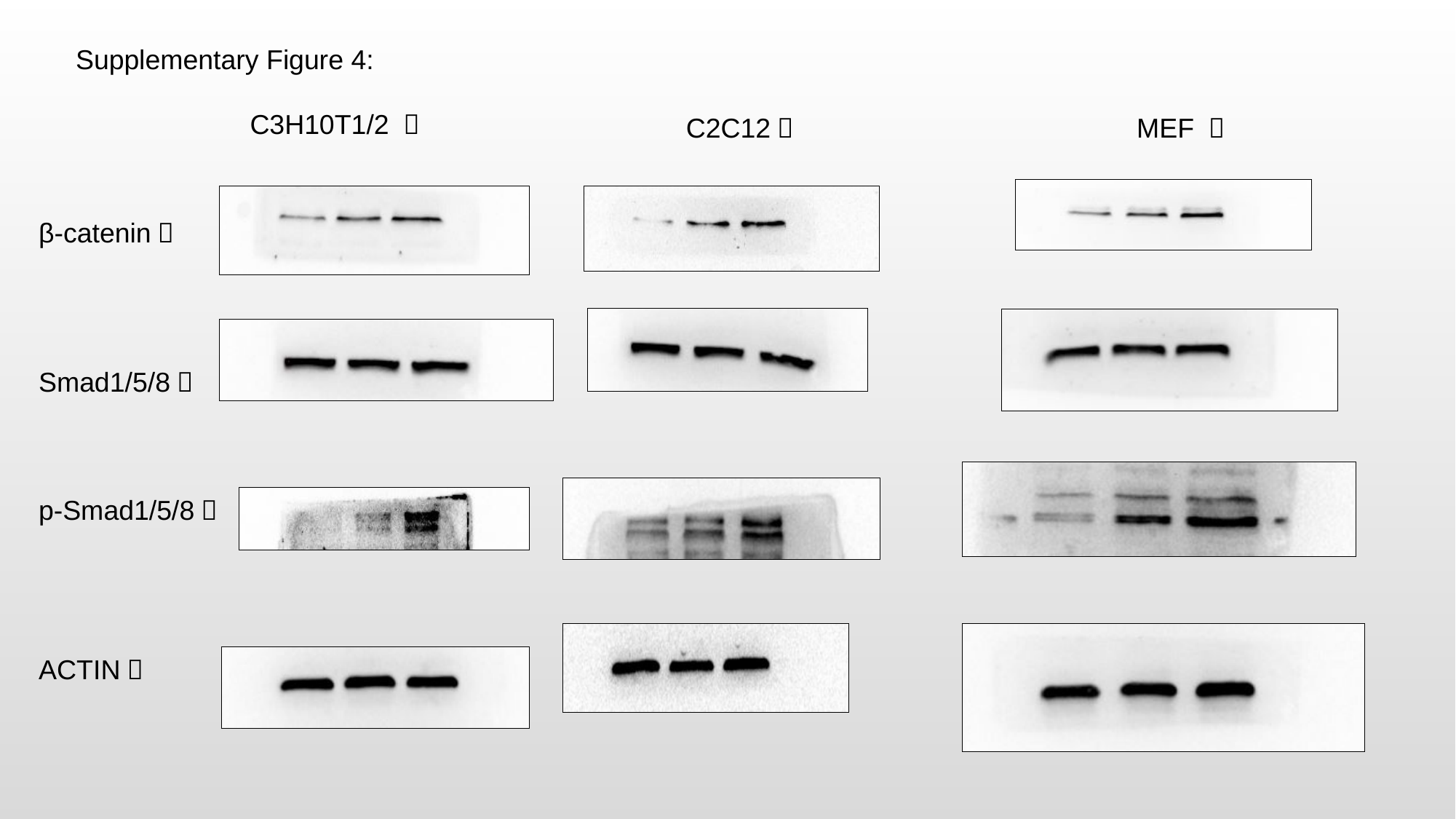

Supplementary Figure 4:
C3H10T1/2 ：
C2C12：
MEF ：
β-catenin：
Smad1/5/8：
p-Smad1/5/8：
ACTIN：

## Slide 14
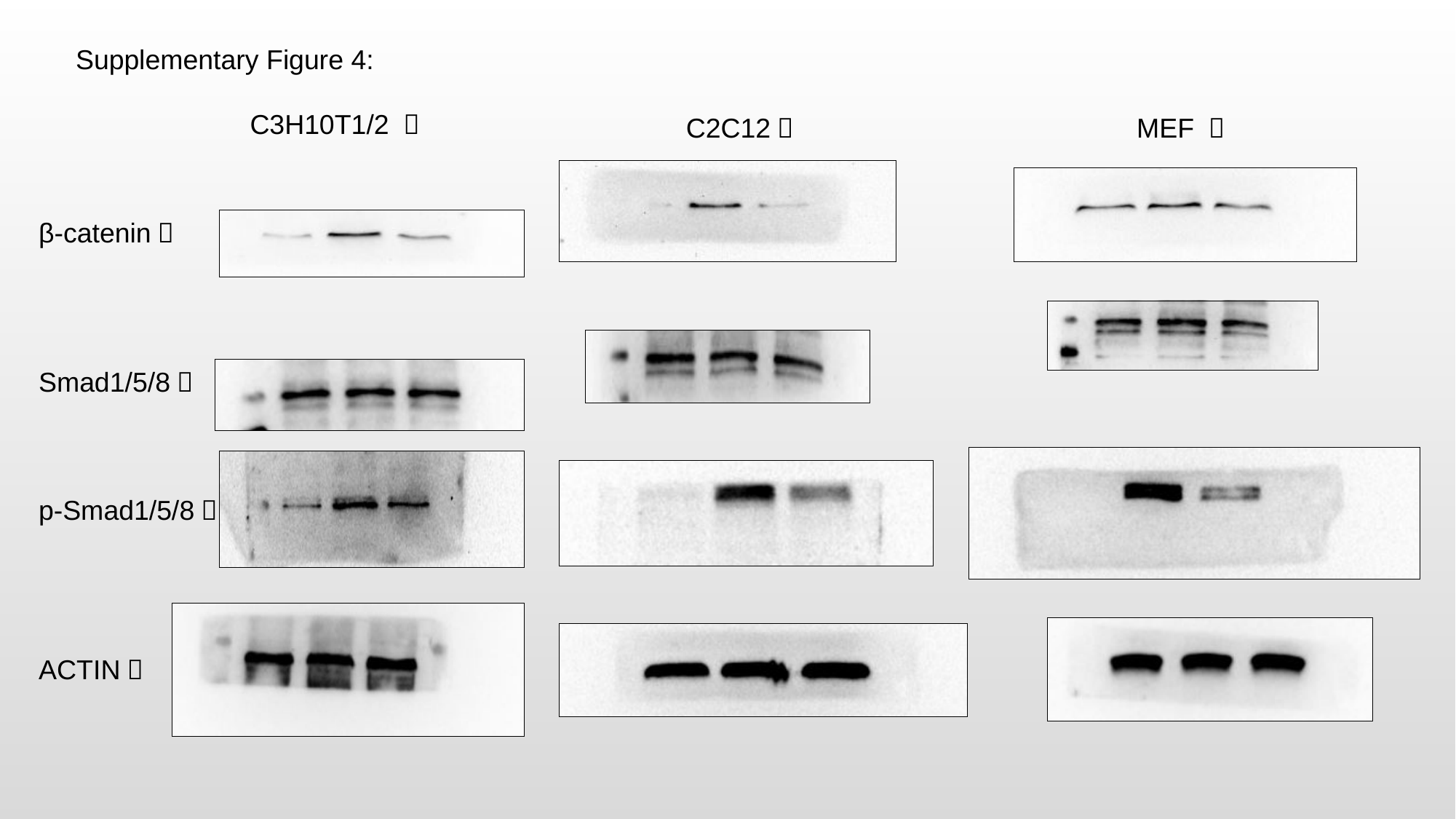

Supplementary Figure 4:
C3H10T1/2 ：
C2C12：
MEF ：
β-catenin：
Smad1/5/8：
p-Smad1/5/8：
ACTIN：

## Slide 15
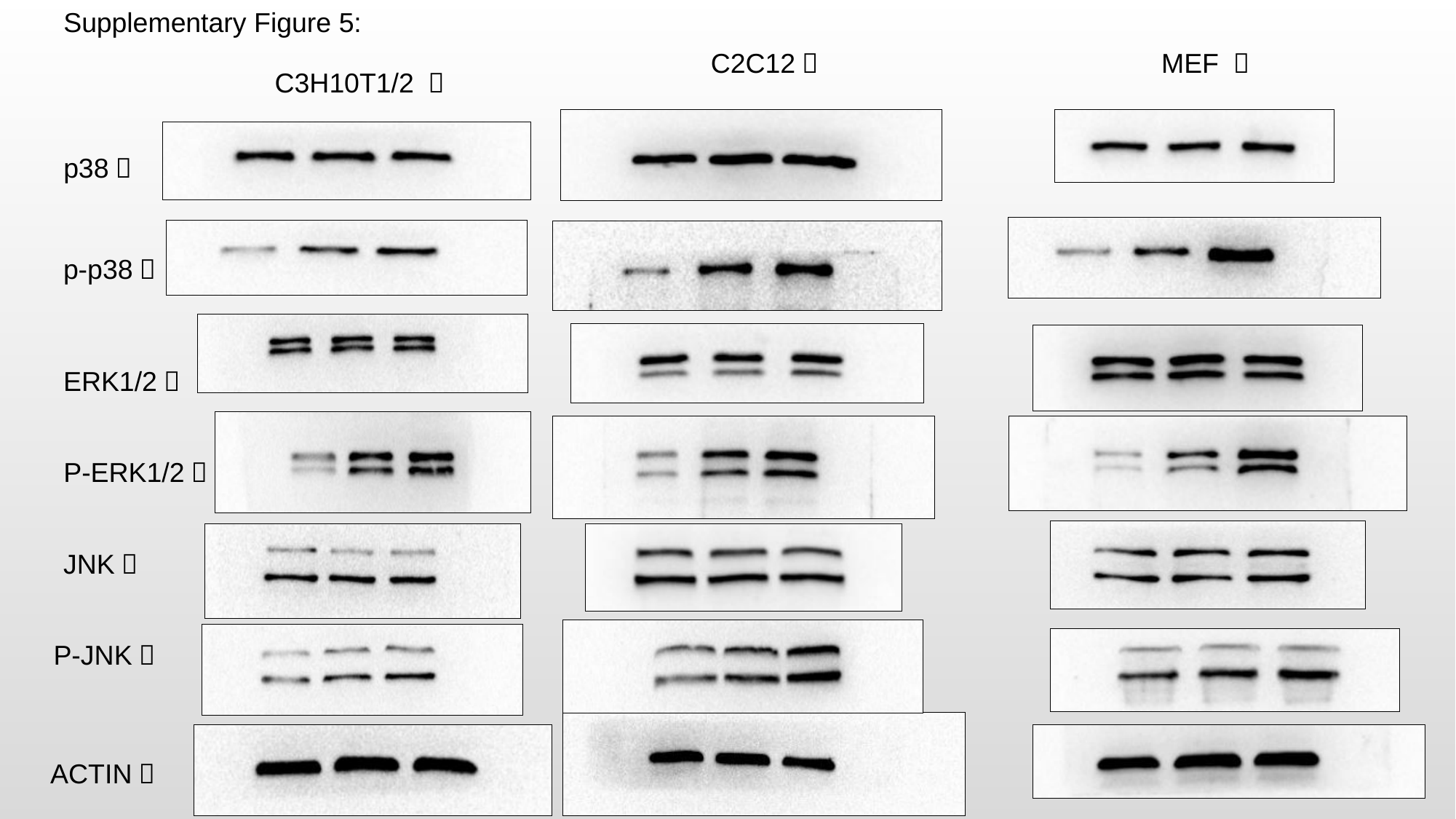

Supplementary Figure 5:
C2C12：
MEF ：
C3H10T1/2 ：
p38：
p-p38：
ERK1/2：
P-ERK1/2：
JNK：
P-JNK：
ACTIN：

## Slide 16
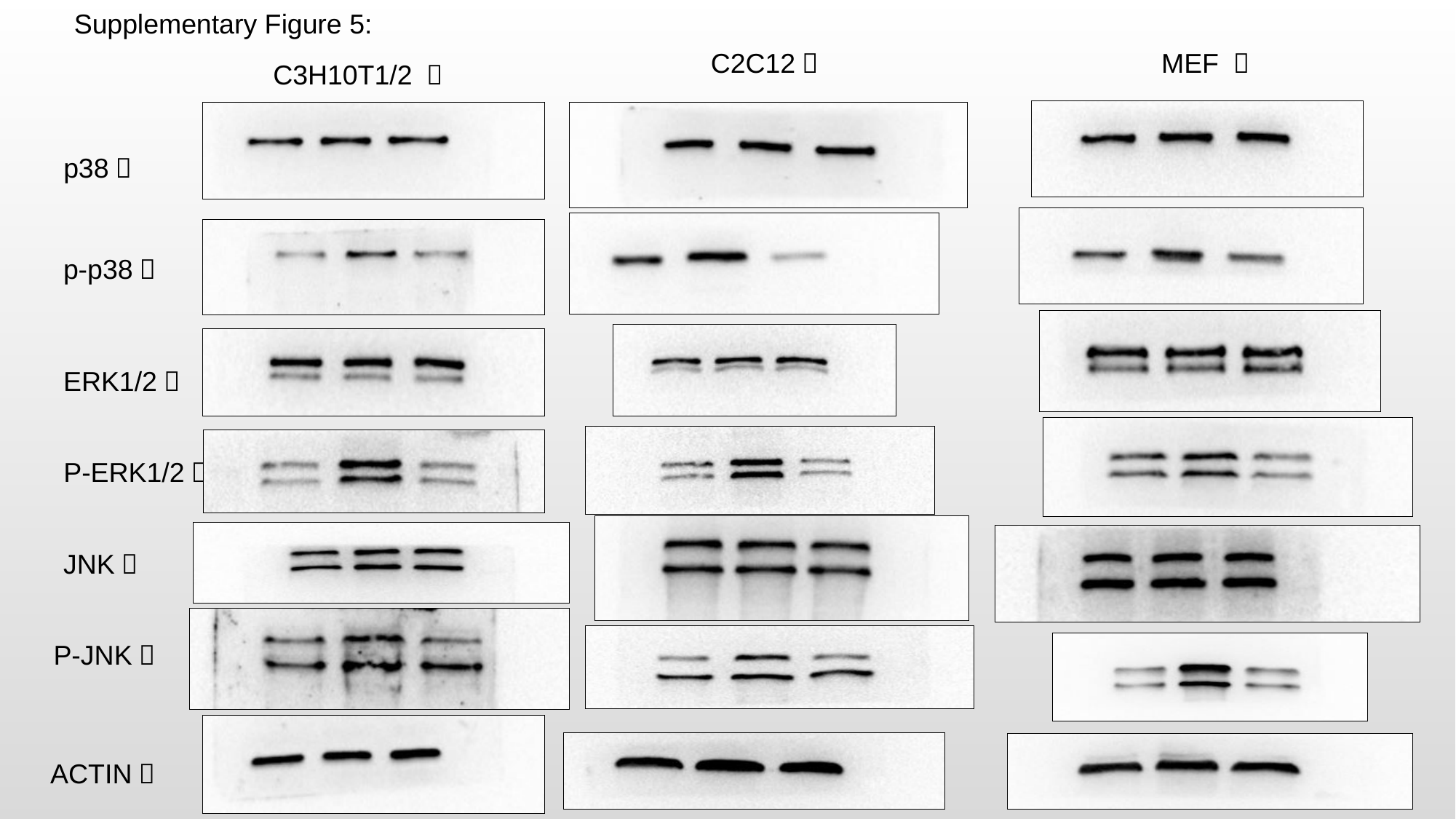

Supplementary Figure 5:
C2C12：
MEF ：
C3H10T1/2 ：
p38：
p-p38：
ERK1/2：
P-ERK1/2：
JNK：
P-JNK：
ACTIN：
